# ATF3‐mediated transactivation of CXCL14 in HSCs during liver fibrosis

**DOI:** 10.1002/ctm2.70040

**Published:** 2024-10-02

**Authors:** Xinmiao Li, Lifan Lin, Yifei Li, Weizhi Zhang, Zhichao Lang, Jianjian Zheng

**Affiliations:** ^1^ Zhejiang Key Laboratory of Intelligent Cancer Biomarker Discovery and Translation, The First Affiliated Hospital of Wenzhou Medical University Wenzhou China

**Keywords:** CXCL14, hepatic stellate cell, liver fibrosis, transcriptional regulation

## Abstract

**Background and aims:**

Myofibroblasts, the primary producers of extracellular matrix, primarily originate from hepatic stellate cells (HSCs), and their activation plays a pivotal role in liver fibrosis. This study aimed to investigate the function of CXC motif ligand 14 (CXCL14) in the progression of liver fibrosis.

**Approach and results:**

CXCL14 knockdown significantly reduced the extent of liver fibrosis. Using Ingenuity pathway analysis and qRT‐PCR, activating transcription factor 3 (ATF3) was identified as a key upstream regulator of CXCL14 expression. Mechanistically, ATF3 was shown to bind to the CXCL14 promoter, promoting its transactivation by TGF‐β in HSCs. Notably, both global CXCL14 deletion (*CXCL14^−/−^*) and HSC/myofibroblast‐specific CXCL14 knockdown significantly attenuated liver fibrosis in mice. RNA‐seq comparisons between *CXCL14^−/−^* and WT mice highlighted Jak2 as the most significantly downregulated gene, implicating its role in the antifibrotic effects of CXCL14 suppression on HSC inactivation. Moreover, Jak2 overexpression reversed the inhibition of liver fibrosis caused by CXCL14 knockdown in vivo.

**Conclusions:**

These findings unveil a novel ATF3/CXCL14/Jak2 signalling axis in liver fibrosis, presenting potential therapeutic targets for the disease.

## Graphical abstract

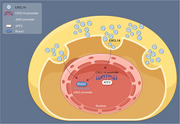

## 1 INTRODUCTION

Chronic liver injury initiates myofibroblast activation in the liver, driving the secretion of extracellular matrix proteins that contribute to fibrous scar formation, ultimately causing liver inflammation and fibrosis.^1^ Hepatic stellate cells (HSCs) are the primary source of these myofibroblasts.^2^ Under normal conditions, quiescent HSCs primarily function as lipid reservoirs and share notable transcriptomic similarities with adipocytes.^3^ In response to liver injury, HSCs are exposed to several profibrogenic factors, including transforming growth factor‐beta (TGF‐β), platelet‐derived growth factor (PDGF) and tumour necrosis factor‐alpha.^4^, ^5^ These factors collectively induce the transdifferentiation of HSCs into myofibroblasts, thus driving the fibrotic response.^6^ As a result, inhibiting HSC activation is a key therapeutic target in managing liver fibrosis.

Chemokines primarily regulate leukocyte migration by inducing chemotaxis, thereby orchestrating inflammatory responses.^7^ Bioinformatics analyses have demonstrated significant upregulation of CXC motif ligand 14 (CXCL14) in a carbon tetrachloride (CCl_4_)‐induced mouse model of fibrosis, highlighting its role as a crucial chemokine in infection and immunity.^8^, ^9^ Antibody neutralisation CXCL14 has been shown to be linked to liver injury, it has been shown to reduce liver injury and steatosis while promoting regeneration.^10^ However, much of the previous research has focused on the upstream mechanisms of CXCL14 in liver fibrosis, leaving its downstream effects and potential clinical applications less explored.^9^

Transcription factors (TFs) are proteins that regulate gene expression by binding selectively to specific DNA sequences.^11^ Several studies have demonstrated the role of TFs in chemokine regulation; for instance, Saliba et al. showed that MAFF regulates CXCL1 levels in myometrial cells.^12^ TFs also play a role in various diseases. For example, Chen et al. found that c‐Myb enhances the invasive potential of hepatocellular carcinoma cells by upregulating osteopontin,^13^ and Winkler et al. discovered that Gata4 deletion induces structural and transcriptional changes in hepatic endothelium, ultimately leading to liver fibrosis.^14^ Due to their wide regulatory capabilities and involvement in many diseases, transcriptional regulators are promising candidates for novel therapeutic targets.^15^, ^16^

This study aimed to investigate the role of CXCL14 in liver fibrosis. Findings revealed that the specific deletion of CXCL14 in HSCs suppressed liver fibrosis in mice via the activating transcription factor 3 (ATF3)/CXCL14/RUNX1/JAK2 signalling pathway. Consequently, CXCL14 was identified as a novel regulator of liver fibrosis with potential therapeutic applications. These results suggest that targeting CXCL14 could offer a promising approach to treating liver fibrosis, enhancing the clinical relevance of these discoveries.

## 2 MATERIALS AND METHODS

### 2.1 Animal

Four‐week‐old mice (15–20 g) were used to establish liver fibrosis models via bile duct ligation (BDL) for 3 weeks or CCl_4_ (50%, 1 µL/g) injections administered twice weekly for 6 weeks. Additionally, mice were treated with anti‐CXCL14 eptides (α‐CXCL14) at a dose of 15 mg/kg. Upon completion of fibrosis modelling, blood, liver tissue and primary cells were collected for subsequent analysis.

CXCL14 knockout (*CXCL14^−/−^*) mice were obtained from Cyagen (Suzhou, China).

Lentivirus encoding shRNA targeting CXCL14 (Lenti‐shCXCL14) and adeno‐associated virus 8 carrying CXCL14‐targeting shRNA (AAV‐shCXCL14), were obtained from Cyagen (Suzhou, China), which were used to construct conditional knockdown (*CXCL14^−/−^ cKd*) mice. Briefly, using adenoviruses or lentiviruses as vectors, construct expression vectors containing the Lrat promoter and shRNA encoding targeting Lenti‐shCXCL14 and AAV‐shCXCL14. Prepare lentiviral and adenoviral particles containing shCXCL14 through cell culture and virus packaging. Finally, obtain a mouse model with *CXCL14^−/−^ cKd* through tail vein injection.

### 2.2 Human specimens

Biopsy samples and sera from patients with cirrhosis were collected from the First Affiliated Hospital of Wenzhou Medical University.

### 2.3 Pathological evaluation of liver

Liver samples were fixed in 10% formalin, embedded in paraffin, sectioned and stained for histological analysis as previously detailed.^17^ Haematoxylin and eosin (HE), Sirius red, and Masson staining were performed, and images were captured using a Leica DM4B microscope.

### 2.4 Immunohistochemistry

As described in a previous study,^18^ liver tissues were fixed in formalin, followed by blocking with 10% BSA and overnight incubation with primary antibodies. Images were subsequently acquired using a Leica DM4B microscope. Details of antibody sources are provided in Table S2.

### 2.5 Isolation of liver cells

Primary HSCs were isolated from the respective groups of mice following previously established protocols.^17^ Cells were cultured in DMEM containing 10% FBS and maintained at 37°C in a humidified environment with 5% CO_2_. Medium were refreshed every 2 days.

Primary hepatocytes and Kupffer cells (KCs) were isolated using a two‐step collagenase liver perfusion method. Mice were sedated, followed by portal vein perfusion with liver perfusion medium to flush out the blood, after which a collagenase solution was infused. The resulting cell suspension was passed through a 70‐µm strainer and centrifuged to separate hepatocytes. The hepatocyte pellet was further purified by centrifugation over a 40% Percoll gradient and cultured in collagen‐coated plates. KCs were isolated from the nonparenchymal fraction.

### 2.6 Cell culture and treatment

The human HSC cell line LX‐2, obtained from Newgainbio (Wuxi, China), was cultured in DMEM with 10% FBS and treated with TGF‐β (2 ng/mL), PDGF (10 ng/mL) or Pacritinib (1 µmol/mL).

### 2.7 Quantitative real‐time PCR (qRT‐PCR)

Total RNA was extracted from cells or liver tissues, and cDNA synthesis was performed using a reverse transcription kit. qRT‐PCR was conducted using the 7500 Fast system, with β‐actin serving as the control for relative quantification of mRNA levels. The 2^−ΔΔCt^ method was applied for mRNA expression analysis, and primer sequences are listed in Table S1.

### 2.8 RNA sequencing (RNA‐seq)

RNA‐seq libraries were prepared using the KAPA Strand RNA‐Seq Library Preparation Kit (Illumina, San Diego, CA, USA). Sequencing was carried out on an Illumina NovaSeq 6000. Transcript expression changes with |Log2 fold change (FC)| > 1.5 and *p* < .05 were considered significant for differential expression analysis.

### 2.9 Western blot

Total protein was extracted and subjected to electrophoresis using 10% SDS‐polyacrylamide gels, followed by transfer to PVDF membranes. After blocking, the membranes were incubated with antibodies. Visualisation was achieved through ECL chemiluminescence, and images were acquired using an automated chemiluminescence imaging system. β‐actin was used as a reference for normalising protein expression, which was quantified via ImageJ software. Antibody sources are detailed in Table S2.

### 2.10 Immunofluorescence staining

For cell and liver specimen staining, cells were fixed, washed, blocked and incubated with antibodies. Liver sections were permeabilised, blocked and incubated with antibodies. Fluorescent antibodies were used for staining, and DAPI was applied to counterstain nuclei. Visualisation was conducted using a fluorescence microscope, with antibody sources listed in Table S2.

### 2.11 5‐Ethyny‐2ʼ‐deoxyuridine (EdU) assay

Cells, after fixation and permeabilisation, were stained with Apollo567 for 30 min, followed by nuclear counterstaining with DAPI. EdU‐positive cells were quantified using a Nikon fluorescence microscope, and ImageJ software was used for analysis.

### 2.12 Chromatin immunoprecipitation (ChIP)

For DNA pull‐down, cells were lysed in SDS buffer and DNA‐fragmented to 100−500 bp using sonication. DNA was immunoprecipitated using specific antibodies or normal mouse IgG. The compound underwent washes with high‐ and low‐salt buffers, and enriched sequences were evaluated by qRT‐PCR.

### 2.13 Luciferase reporter assay

Promoter regions of wild‐type (Jak2‐WT) and mutant‐type (Jak2‐MUT) constructs were cloned into pGLO plasmids. HEK293 cells were transfected with these constructs using Lipofectamine 3000. Relative luciferase activity was measured 48 h post‐transfection using the Luciferase Assay Kit, with Renilla luciferase serving as the internal control for normalisation.

### 2.14 Statistical analysis

Statistical analyses were conducted using Prism 10, with data expressed as mean ± standard deviation. Differences between two groups were assessed using Student's *t*‐test, and one‐way analysis of variance (ANOVA) was employed for multiple group comparisons. Statistical significance was set at *p* < .05.

## 3 RESULTS

### 3.1 Elevated CXCL14 during HSC activation

**FIGURE 1:**
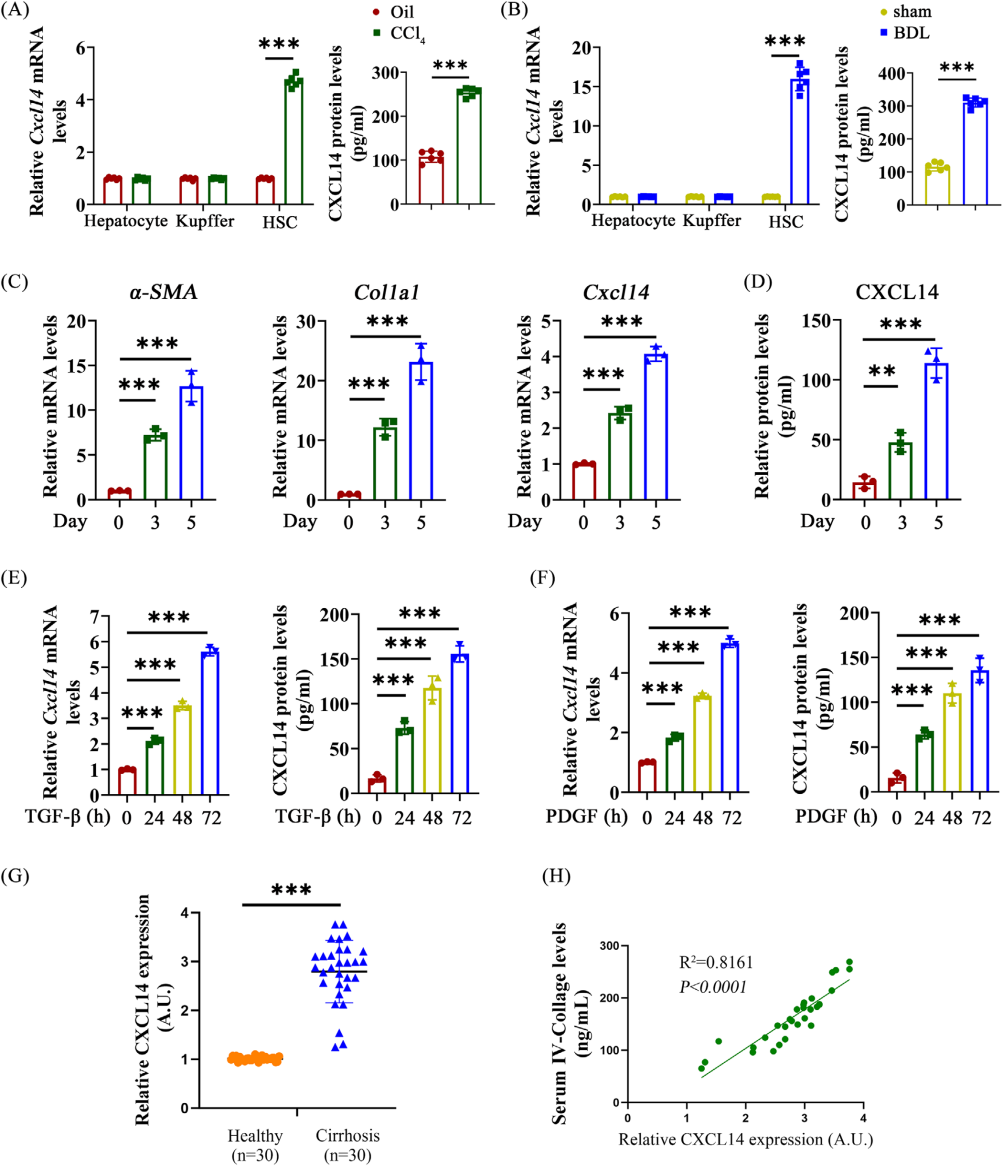
Elevated CXCL14 during HSC activation. Primary HSCs were isolated and cultured in vitro for spontaneous activation. (A, B) Hepatocytes, KCs and primary HSCs were isolated from CCl_4_‐ and BDL‐induced liver fibrosis mice, and CXCL14 expression was evaluated by qRT‐PCR and ELISA. (C) mRNA levels of α‐SMA, Col1a1 and CXCL14 in primary HSCs. (D) CXCL14 levels in primary HSCs detected by ELISA. (E, F) CXCL14 expression in LX‐2 cells measured by qRT‐PCR and ELISA following TGF‐β (2 ng/mL) or PDGF (10 ng/mL) treatment. (G) CXCL14 expression in liver tissues of healthy individuals and patients with cirrhosis, assessed by qRT‐PCR. (H) Correlation between CXCL14 and serum IV‐Collagen levels analysed by linear regression (*n* = 30). **p < .01, ***p < .001.

To investigate the regulatory role of CXCL14 in liver fibrosis pathogenesis, CXCL14 levels were first measured in primary cells isolated from the CCl_4_ and oil groups. qRT‐PCR analysis revealed that CXCL14 was significantly elevated in HSCs compared to hepatocytes and KCs (Figure 1A). Similarly, ELISA results showed a marked increase in CXCL14 expression in HSCs from the CCl_4_ group compared to the oil group (Figure 1A). This elevation was also observed in another liver fibrosis model, the BDL model (Figure 1B). In primary HSCs isolated from C57/BL6 mice during spontaneous activation, CXCL14 expression increased alongside higher levels of myofibroblast markers such as α‐SMA and Col1A1 (Figure 1C and D). Consistent with these findings, LX‐2 cells treated with profibrotic factors TGF‐β or PDGF exhibited a time‐dependent rise in CXCL14 levels (Figure 1E and F). Moreover, qRT‐PCR analysis of liver specimens from patients with cirrhosis and healthy donors demonstrated significantly elevated CXCL14 levels in cirrhotic individuals (Figure 1G). This increase correlated with fibrosis markers (Figure 1H), suggesting a potential role for CXCL14 in the pathogenesis of liver fibrosis and cirrhosis. These results indicate that HSC activation is closely associated with increased CXCL14 expression.

### 3.2 ATF3 mediates TGF‐β/PDGF‐induced CXCL14 transcription in HSCs

**FIGURE 2:**
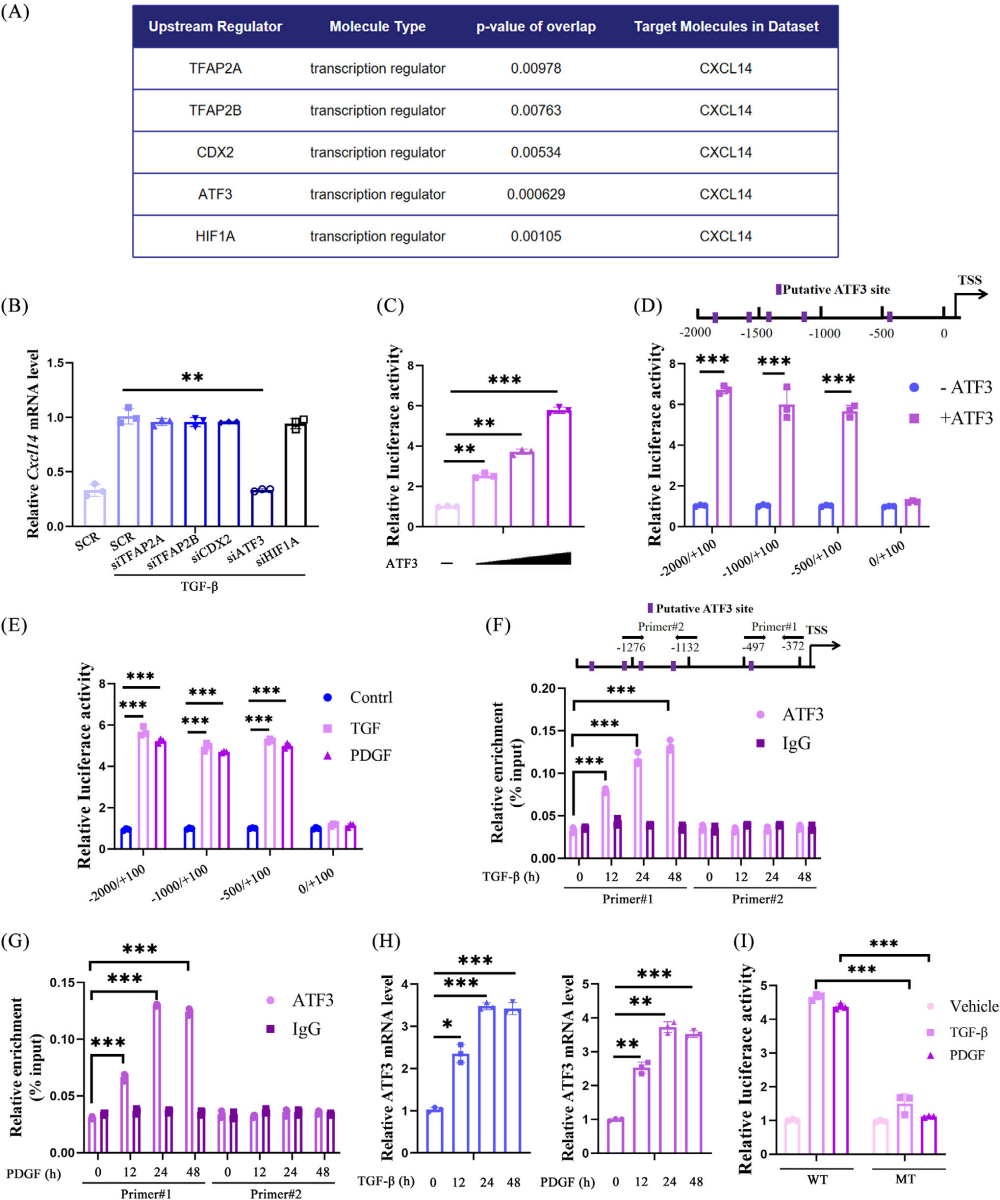
ATF3 mediates TGF‐β/PDGF‐induced CXCL14 transcription in HSCs. (A) IPA analysis. (B) mRNA levels of CXCL14 in LX‐2 cells transfected with specific siRNAs, followed by TGF‐β treatment. (C) Luciferase activity analysis of a CXCL14 promoter‐luciferase construct transfected into LX‐2 cells with increasing doses of ATF3. (D, E) Luciferase activity of various CXCL14 promoter‐luciferase constructs transfected into LX‐2 cells with or without ATF3, TGF‐β or PDGF. (F, G) ChIP analysis of LX‐2 cells treated with TGF‐β or PDGF. (H) mRNA levels of ATF3 in LX‐2 cells following TGF‐β or PDGF treatment. (I) Luciferase activity of WT and MT CXCL14 promoter‐luciferase constructs transfected into LX‐2 cells followed by TGF‐β or PDGF treatment. Each value represents the mean ± SD of three experiments. *p < .05, **p < .01, ***p < .001.

Next, the regulatory mechanisms underlying CXCL14 induction during HSC activation were examined. Ingenuity Pathway Analysis (IPA) identified several potential upstream TFs involved in CXCL14 regulation (Figure 2A). siRNA‐mediated knockdown of these TFs revealed that only ATF3 depletion significantly reduced CXCL14 induction by TGF‐β in LX‐2 cells (Figure 2B). Dose‐dependent overexpression of ATF3 markedly enhanced CXCL14 promoter activity, as demonstrated by luciferase reporter assays (Figure 2C). Serial deletions of the CXCL14 promoter‐luciferase construct indicated that ATF3 overexpression, as well as TGF‐β and PDGF treatments, required the presence of a binding motif located between −500 and 0 relative to the transcription start site for reporter activation (Figure 2D and E). ChIP assays confirmed that TGF‐β and PDGF treatments increased ATF3 binding to the −497/−372 region near the CXCL14 promoter, but not to a more distal region (Figure 2F and G). This enhanced binding may be attributed to the upregulation of ATF3 itself in response to TGF‐β or PDGF (Figure 2H). Notably, mutating the proximal ATF3 binding site completely abolished the reporter's response to TGF‐β and PDGF (Figure 2I). Collectively, these results suggest that ATF3 plays a pivotal role in regulating CXCL14 transcription in HSCs.

### 3.3 CXCL14 regulates HSC activation in vitro

**FIGURE 3:**
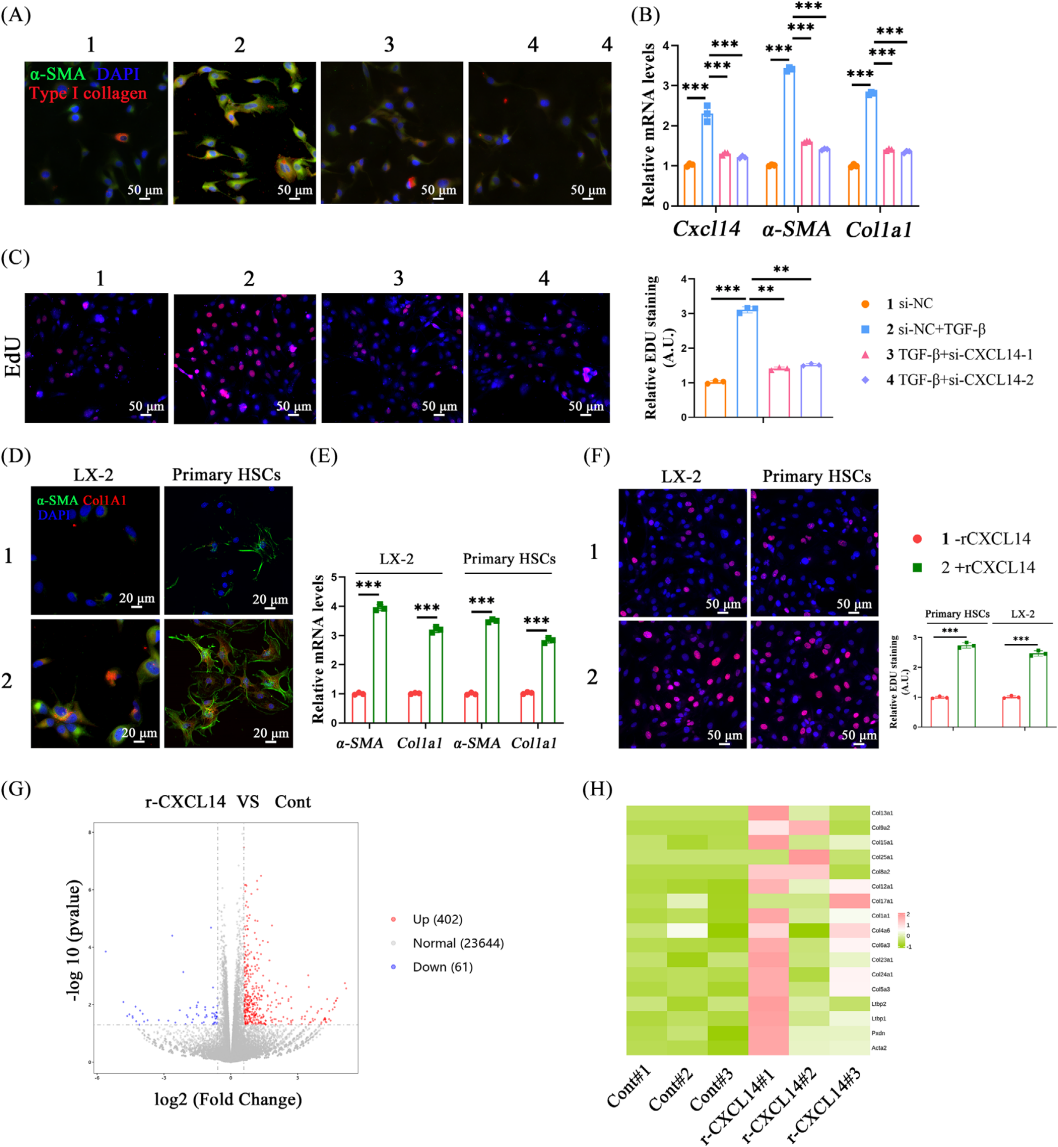
CXCL14 regulates HSC activation in vitro. Primary HSCs were isolated and cultured for spontaneous activation. (A) Immunofluorescence staining for α‐SMA (green) and Type I collagen (red) in LX‐2 cells transfected with indicated siRNAs and treated with TGF‐β. (B) mRNA levels of CXCL14, α‐SMA and Col1a1 in LX‐2 cells. (C) EdU assay in LX‐2 cells. (D) Immunofluorescence staining for α‐SMA (green) and CXCL14 (red) in LX‐2 cells and primary HSCs treated with rCXCL14 (50 ng/mL). (E) mRNA levels of α‐SMA and CXCL14 in LX‐2 cells and primary HSCs treated with rCXCL14 (50 ng/mL). (F) EdU assay in LX‐2 cells and primary HSCs. (G, H) Volcano plots and heatmap of primary HSCs treated with rCXCL14 (50 ng/mL), followed by RNA‐seq analysis. Each value represents the mean ± SD of three experiments. **p < .01, ***p < .001.

The role of CXCL14 in mediating HSC activation in vitro was explored by designing siRNA to knock down endogenous CXCL14 in LX‐2 cells. qRT‐PCR and immunofluorescence analyses demonstrated that CXCL14 suppression effectively inhibited TGF‐β‐induced upregulation of α‐SMA and Col1A1 (Figure 3A and B). Edu analysis further revealed that the proliferation of LX‐2 cells stimulated by TGF‐β was markedly reduced in the absence of CXCL14 (Figure 3C). In contrast, treatment with recombinant CXCL14 (rCXCL14) significantly elevated myofibroblast marker levels, as shown by qRT‐PCR and immunofluorescence (Figure 3D and E), and increased HSC proliferation, as confirmed by Edu analysis (Figure 3F). RNA‐seq analysis of primary HSCs treated with rCXCL14 identified 402 upregulated and 61 downregulated genes (fold change > 1.5, *p* < .05) (Figure 3G). Further investigation revealed that many of the upregulated genes were associated with profibrotic processes (Figure 3H). These results collectively indicate that CXCL14 plays a regulatory role in HSC activation.

### 3.4 CXCL14 deficiency alleviates liver fibrosis in mice

**FIGURE 4:**
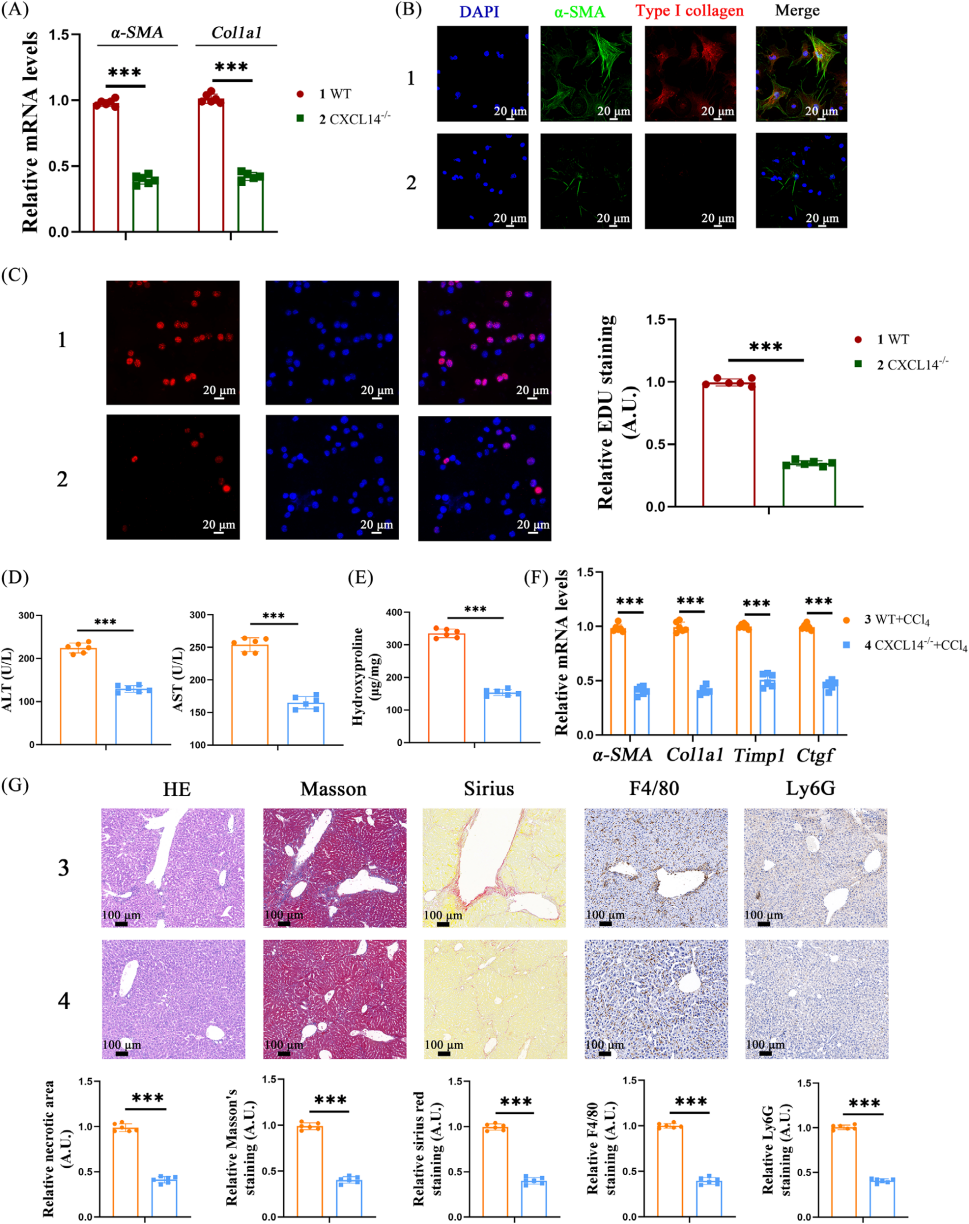
CXCL14 deficiency alleviates liver fibrosis in mice. Primary HSCs were isolated and cultured for spontaneous activation. (A) mRNA levels of α‐SMA and Col1a1 in primary HSCs isolated from WT and *CXCL14^−/−^* mice. (B) Immunofluorescence staining for α‐SMA (green) and Type I collagen (red) in primary HSCs. (C) EdU assay. (D–G) WT and CXCL14 knockout mice were injected with CCl_4_ for 6 weeks. (D, E) Levels of ALT, AST and Hyp. (F) mRNA levels of profibrogenic genes α‐SMA, Col1a1, Timp1 and Ctgf in liver tissues from WT and *CXCL14^−/−^* mice after CCl_4_ treatment. (G) HE, Masson, Sirius Red, F4/80 and Ly6G staining. Each value represents the mean ± SD of six experiments. ***p < .001.

Primary HSCs were isolated from *CXCL14^−/–^* and wild‐type (WT) mice, followed by spontaneous activation in vitro. qRT‐PCR, immunofluorescence, and Edu assays showed that CXCL14 knockout resulted in HSC inactivation, characterised by reduced expression of myofibroblast markers and decreased cell proliferation (Figure 4A–C). To evaluate the impact of CXCL14 loss on liver fibrosis progression, *CXCL14^−/−^* mice were treated with CCl_4_. Compared to WT controls, *CXCL14^−/−^* mice exhibited improved liver function, as evidenced by lower ALT/AST levels (Figure 4D). CXCL14 inhibition also led to a reduction in hydroxyproline (Hyp) content and myofibroblast marker expression (Figure 4E and F). Histological analyses using HE, Masson, Sirius red, and other staining methods revealed a significant decrease in collagen deposition in *CXCL14^−/−^* mice. Additionally, reduced infiltration of F4/80^+^ macrophages and Ly6G^+^ neutrophils was observed (Figure 4G). Consistent with these results, *CXCL14^−/−^* mice subjected to the BDL model showed a marked reduction in liver fibrosis (Figure S1). These data underscore the critical role of CXCL14 in driving HSC activation and liver fibrosis.

**FIGURE 5:**
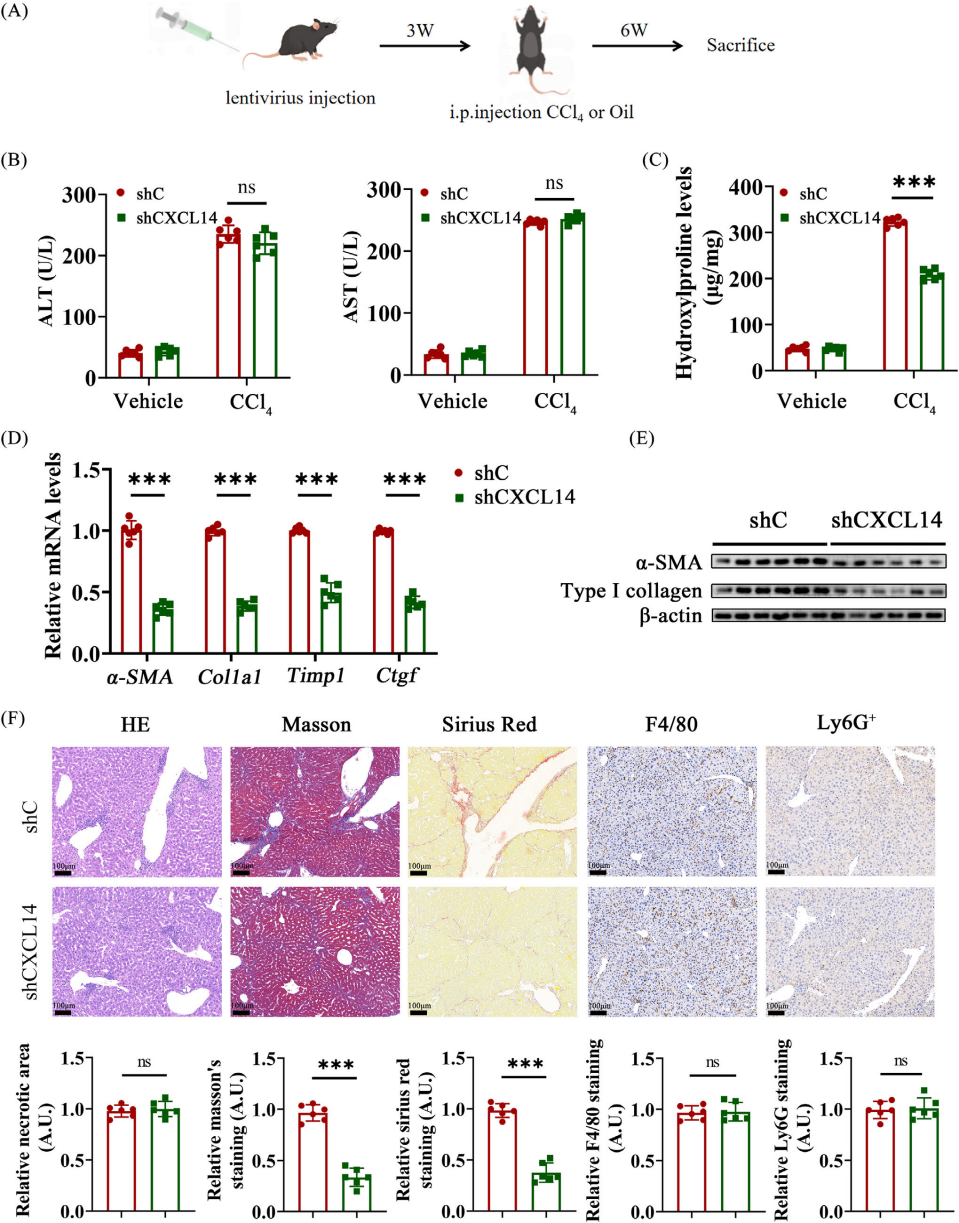
HSC‐specific CXCL14 deletion mitigates liver fibrosis in mice Lenti‐shCXCL14 or Lenti‐shC were injected into C57/BL6 mice, followed by CCl_4_ injections for 6 weeks. (A) Experimental design. (B, C) Levels of ALT, AST and Hyp. (D) mRNA levels of α‐SMA, Col1a1, Timp1 and Ctgf in liver tissues. (E) Protein levels of α‐SMA and Type I collagen in liver tissues. (F) HE, Masson, Sirius Red, F4/80 and Ly6G staining. Each value represents the mean ± SD of six experiments. ***p < .001; ns, not significant.

Given the predominant expression of CXCL14 in HSCs during liver fibrosis (Figure 1A), this study aimed to determine whether selectively depleting CXCL14 in HSCs could alleviate liver fibrosis. A Lenti‐shCXCL14, placed downstream of the LRAT promoter/enhancer sequence, was constructed.^19^ qRT‐PCR confirmed that Lenti‐shCXCL14 effectively reduced CXCL14 expression specifically in HSCs, with no detectable changes in hepatocytes, KCs, liver sinusoidal endothelial cells (LSECs), or neutrophils (Figure S2A). C57/BL6 mice were then injected with either Lenti‐shCXCL14 or control lentivirus (shC) via the tail vein, followed by treatment with CCl_4_ or oil (Figure 5A). While specific CXCL14 depletion in HSCs did not significantly affect CCl_4_‐induced liver injury markers, as indicated by similar ALT and AST levels (Figure 5B), a notable reduction in liver hyp content was observed (Figure 5C). Additionally, decreased mRNA and protein levels of myofibroblast markers were detected (Figure 5D and E). Histological analysis revealed reduced collagen deposition in shCXCL14‐treated mice, as shown by HE, Masson and Sirius red staining, although CXCL14 deficiency did not influence immune cell infiltration in the CCl_4_ model (Figure 5F). Comparable results were obtained in mice subjected to the BDL procedure (Figure S3).

Further investigation into the anti‐fibrotic effect of CXCL14 knockdown was conducted using AAV‐shCXCL14, driven by the Postn promoter to silence CXCL14 in mature myofibroblasts.^20^ qRT‐PCR demonstrated that AAV‐shCXCL14 treatment significantly reduced CXCL14 expression in myofibroblasts (activated HSCs) without affecting other cell types (Figure S2B). In reduction of CXCL14 mice, liver fibrosis was notably suppressed without impacting liver injury markers (ALT/AST) or immune infiltration in both CCl_4_ and BDL models (Figures S4 and S5). Collectively, these results suggest that inhibiting CXCL14 plays a key role in mitigating liver fibrosis.

### 3.5 Jak2 is involved in the role of CXCL14 in HSC activation

**FIGURE 6:**
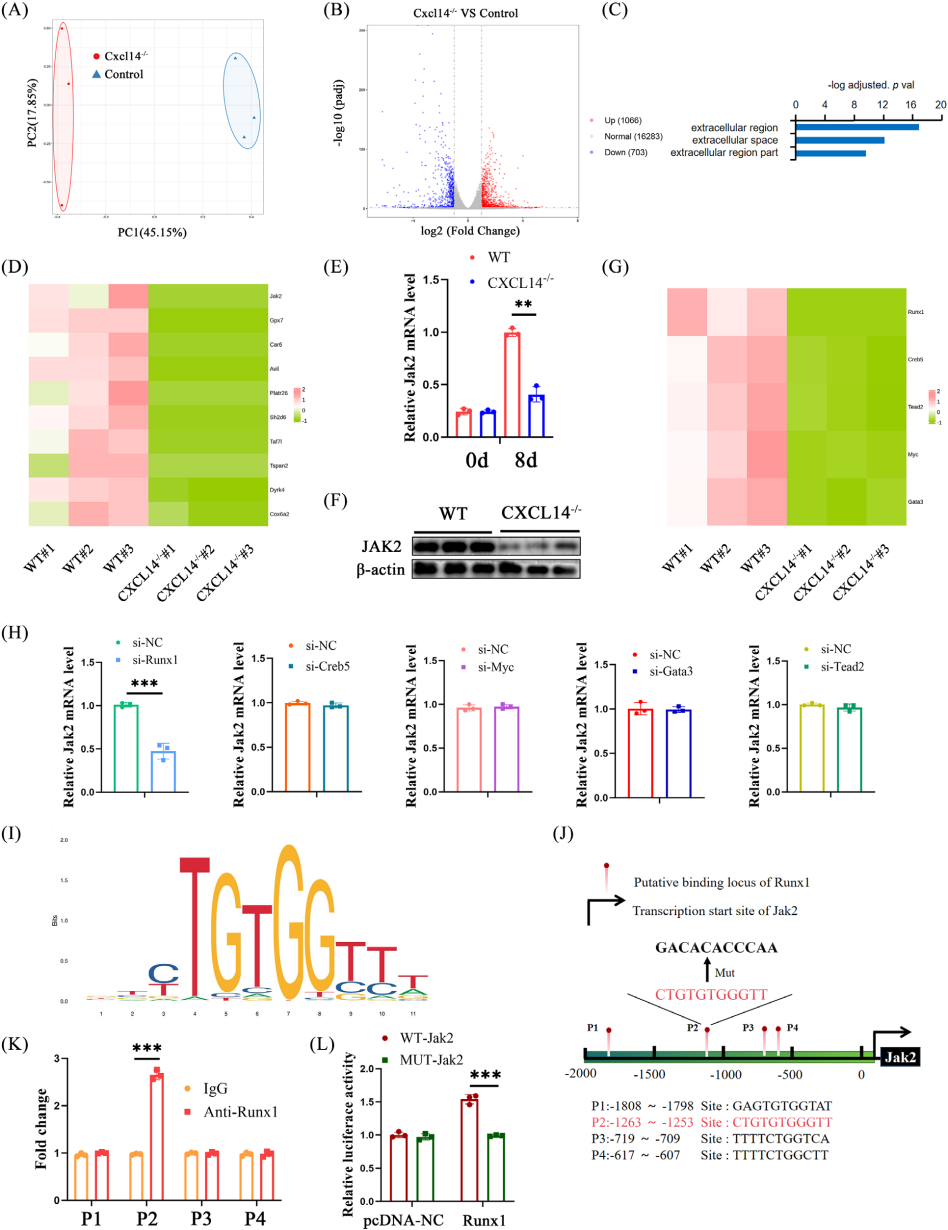
Jak2 is involved in CXCL14‐mediated HSC activation. Primary HSCs were isolated from WT and *CXCL14^−/−^* mice, cultured for spontaneous activation, and analysed by RNA‐seq. (A) PCA plot. (B) Volcano plot. (C) GO analysis. (D) Heatmap of differentially expressed genes. (E, F) mRNA and protein levels of Jak2 in primary HSCs. (G) Heatmap of TFs. (H) mRNA levels of Runx1, Creb5, Myc, Gata3 and Tead2. (I) Runx1‐binding sites in the Jak2 promoter predicted by JASPAR (http://jaspar.genereg.net/). (J) Schematic of potential Runx1 binding sites in the Jak2 promoter. (K) ChIP analysis of Runx1 occupancy at the Jak2 promoter. (L) Luciferase reporter assays. Each value represents the mean ± SD of three experiments. **p < .01, ***p < .001.

RNA‐seq was subsequently conducted to examine differentially expressed transcriptomes in isolated HSCs between *CXCL14^−/−^* and WT mice, aiming to uncover the mechanism by which CXCL14 inhibition mediates HSC inactivation. Principal component analysis (PCA) demonstrated a distinct transcriptomic shift due to CXCL14 deficiency (Figure 6A). A volcano plot revealed 1066 upregulated and 703 downregulated genes in the *CXCL14^−/−^* group (Figure 6B). Gene Ontology (GO) analysis indicated that pathways associated with myofibroblast maturation were involved in CXCL14 inhibition's impact on HSC activation (Figure 6C). Notably, Jak2 was the most significantly downregulated gene in the *CXCL14 ^‐/−^* group (Figure 6D). Both in vivo and in vitro, CXCL14 knockdown or depletion reduced Jak2 levels (Figures 6E and F, S6C and D, and S7), whereas rCXCL14 treatment elevated Jak2 expression in vitro (Figure S6A and B). Additionally, RNA‐seq TF analysis identified five TFs with the most significant differential expression for further investigation (Figure 6G). qRT‐PCR analysis of Jak2 mRNA levels following TF knockdown revealed that only Runx1 deficiency significantly decreased Jak2 expression in primary HSCs (Figure 6H). In contrast, inhibition of Creb5, Myc, Gata3 and Tead2 did not alter Jak2 expression. Further exploration of Runx1's interaction with Jak2 revealed four potential Runx1 binding motifs (P1‐P4) within the Jak2 promoter, identified via JASPAR (Figure 6I and J). ChIP‐qPCR confirmed that the P2 motif was likely the Runx1 binding site (Figure 6K). Luciferase reporter assays demonstrated that Runx1 upregulated Jak2‐WT but not Jak2‐MuT (with a mutated P2 site), confirming the interaction at the P2 site (Figure 6L). Collectively, these results suggest that CXCL14 regulates Jak2 expression partially through Runx1.

### 3.6 CXCL14 promotes HSC activation as well as liver fibrosis via Jak2

**FIGURE 7:**
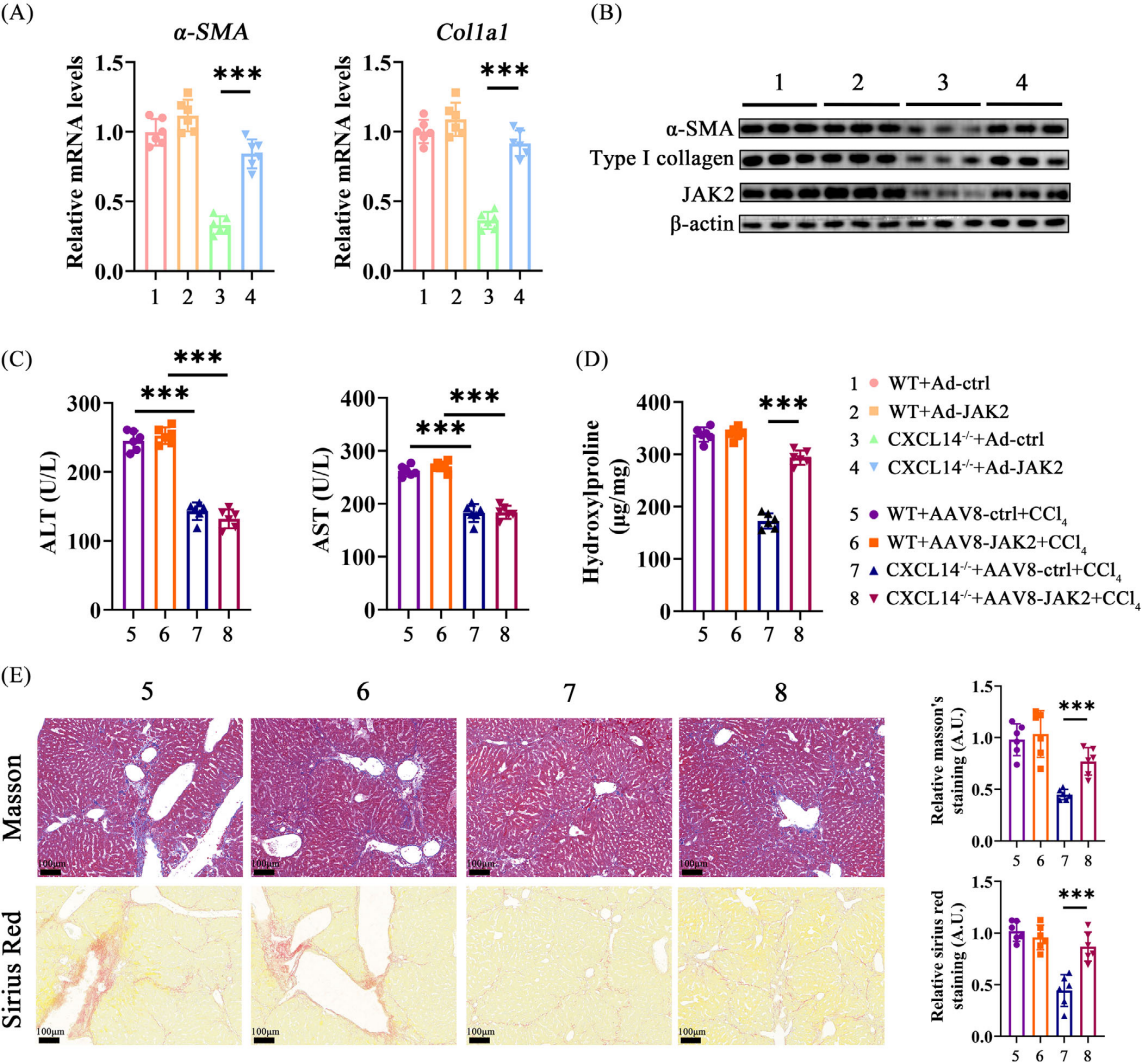
CXCL14 promotes HSC activation and liver fibrosis via Jak2. (A, B) Primary HSCs were isolated from WT and *CXCL14^−/−^* mice and transduced with adenovirus carrying a Jak2 expression vector (Ad‐Jak2) or control vector (Ad‐ctrl). (A) mRNA levels of α‐SMA and Col1a1 in primary HSCs. (B) Protein levels of α‐SMA, Type I collagen, and Jak2 in primary HSCs. (C–E) AAV8 carrying a Jak2 expression vector (AAV8‐Jak2) or control vector (AAV8‐ctrl) was injected into WT and *CXCL14^−/−^* mice, followed by CCl_4_ injection for 6 weeks. (C, D) Levels of ALT, AST and Hyp. (E) Masson and Sirius Red staining. Each value represents the mean ± SD of six experiments. ***p < .001.

To further elucidate the functional relationship between CXCL14 and Jak2, we investigated whether CXCL14 promotes HSC activation and liver fibrosis through Jak2. qRT‐PCR results indicated that Jak2 knockdown or Pacritinib (a Jak2 inhibitor) blocked the upregulation of myofibroblast marker genes in HSCs induced by rCXCL14 (Figure S8). Conversely, overexpression of Jak2 via an adenoviral vector restored myofibroblast marker levels in HSCs from the *CXCL14^−/−^* group (Figure 7A and B). To test whether reintroducing Jak2 would rescue the fibrogenic response in *CXCL14^−/−^* mice, AAV8 carrying a Jak2 expression vector was administered after CCl_4_ injection. Notably, Jak2 overexpression did not affect liver injury markers in *CXCL14^−/−^* mice treated with CCl_4_ (Figure 7C). However, Jak2 overexpression reversed the suppression of liver fibrosis due to CXCL14 knockdown, as evidenced by hyp quantification, Masson, and Sirius red staining (Figure 7D and E). Similarly, Jak2 overexpression mitigated the effects of CXCL14 deficiency in a BDL‐induced liver fibrosis model (Figure S9). In summary, these results indicate that CXCL14 facilitates HSC activation and liver fibrosis through the Jak2 pathway.

### 3.7 Inhibition CXCL14 ameliorates liver fibrosis in vivo

**FIGURE 8:**
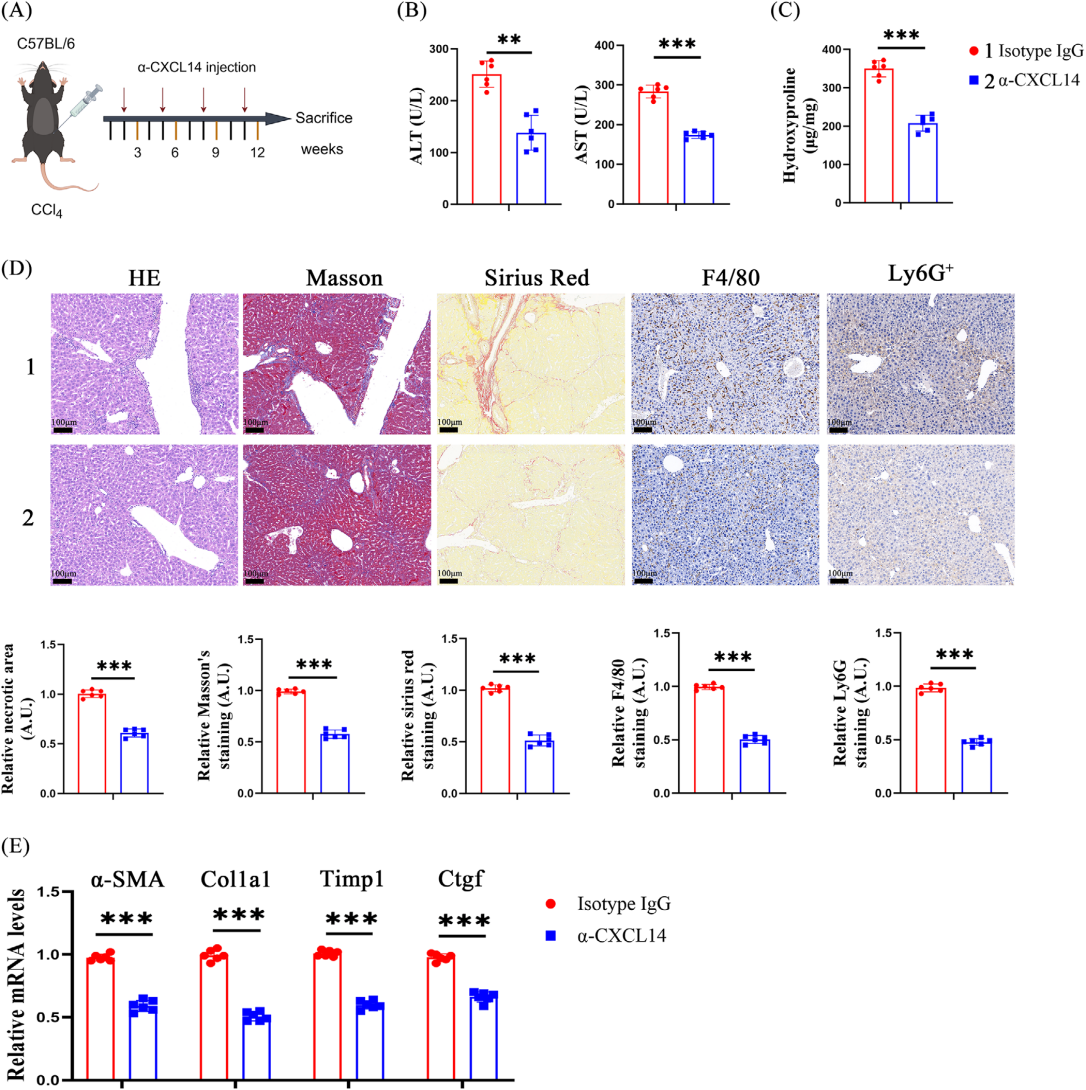
Inhibition of CXCL14 ameliorates liver fibrosis in vivo. CCl_4_‐induced liver fibrosis mice were treated with α‐CXCL14 (15 mg/kg) or isotype IgG (control) every 3 weeks for 12 weeks. (A) Experimental design. (B, C) Levels of ALT, AST and Hyp. (D) HE, Masson, Sirius Red, F4/80 and Ly6G staining. (E) mRNA levels of α‐SMA, Col1a1, Timp1 and Ctgf. Each value represents the mean ± SD of six experiments. **p < .01, ***p < .001.

The role of CXCL14 interference in the suppression of liver fibrosis was investigated. Mice were first treated with CCl_4_ and subsequently injected with α‐CXCL14 (Figure 8A). Neutralisation of CXCL14 resulted in reduced liver injury and diminished immune cell infiltration (Figure 8B and D). Furthermore, α‐CXCL14 administration decreased collagen expression and downregulated myofibroblast marker levels (Figure 8C–E). Consistent findings were observed in the BDL model, where α‐CXCL14 treatment improved liver injury, reduced hepatic immune infiltration, and alleviated liver fibrosis (Figure S10). These results collectively indicate that reduction of CXCL14 attenuates liver fibrosis, at least in part, by inhibiting CXCL14.

## 4 DISCUSSION

The transition of HSCs into myofibroblasts is pivotal in the progression of liver fibrosis.^21^ This study reveals a regulatory mechanism involving CXCL14, where the use of antagonists or blocking antibodies against CXCL14 led to a significant reduction in liver fibrosis in established animal models. This is similar to the findings of Liao et al.^22^ These results highlight CXCL14's regulatory role and suggest its potential as a therapeutic target for liver fibrosis.

Our results demonstrate that CXCL14 is specifically upregulated in HSCs during liver fibrosis, with no corresponding induction in hepatic parenchymal cells. The mechanism underlying this selective expression remains unclear. One possibility is that the transcriptional regulator ATF3, an upstream modulator of CXCL14, is preferentially enriched in HSCs or myofibroblasts. ATF3 has been implicated in the progression of liver fibrosis,^23^ but further research is necessary to elucidate the precise regulatory pathways by which ATF3 influences fibrosis.

RNA‐sequencing data reveal that exogenous CXCL14 stimulation promotes a myofibroblast‐like phenotype in HSCs, while CXCL14 deletion mitigates this signature. The attenuation of liver fibrosis in *CXCL14^−/−^ cKd* mice suggests that CXCL14's profibrogenic effects are primarily mediated through its intrinsic action on HSCs. While several chemokines have been implicated in liver fibrosis,^24^ most appear to exert their effects through myeloid‐derived cells, modifying the liver's immune microenvironment. For instance, Berres et al. demonstrated that transplanting bone marrow from WT mice into CCL5 knockout mice restored liver fibrosis, potentially by normalising macrophage infiltration.^25^ Interestingly, systemic CXCL14 deletion resulted in concurrent reductions in liver injury markers and fibrosis, evidenced by decreased AST and ALT levels, along with reduced F4/80 and LY6G markers. This suggests that the absence of CXCL14 limits liver injury and fibrosis, possibly by inhibiting inflammatory cell infiltration. However, *CXCL14^−/−^ cKd* did not significantly alter liver injury markers, reinforcing the hypothesis that HSC‐derived CXCL14 operates primarily in a paracrine or autocrine fashion to promote HSC‐to‐myofibroblast transformation. Meanwhile, CXCL14 from other cellular sources may regulate immune cell trafficking and contribute to the proinflammatory microenvironment driving liver injury. Further research is essential to clarify these mechanisms in future studies.

RNA‐seq screening, coupled with transcriptional and functional assays, identified Jak2 as a critical mediator of the fibrogenic response induced by CXCL14, establishing it as a direct downstream target. This discovery aligns with previous studies that highlight Jak2's significant role in liver fibrosis, where both animal models and human tissue analyses have demonstrated Jak2 upregulation.^26^ The development of specific Jak2‐targeting antibodies as a therapeutic approach for liver fibrosis warrants further investigation, potentially enhancing the translational relevance of these findings.

In conclusion, our results provide strong evidence supporting the critical role of CXCL14 in driving the transformation of HSCs into myofibroblasts and the progression of liver fibrosis. Moreover, targeting the CXCL14‐Jak2 signalling axis presents a promising therapeutic avenue for the treatment of liver fibrosis.

## AUTHOR CONTRIBUTIONS

The study was conceptualised by Jianjian Zheng. Xinmiao Li, Weizhi Zhang and Lifan Lin carried out the majority of the experiments. Xinmiao Li, Zhichao Lang and Yifei Li analysed the data and prepared the initial draft of the manuscript, with subsequent revisions. All authors contributed to the work, reviewed the final manuscript and approved its submission.

## CONFLICT OF INTEREST STATEMENT

The authors declare no financial conflicts of interest or personal relationships that could have influenced the findings presented in this paper.

## ETHICS STATEMENT

All procedures involving human samples followed the principles of the Declaration of Helsinki and were approved by the Ethics Committee of the First Affiliated Hospital of Wenzhou Medical University (KY2021‐177) on 18 November 2021. The Laboratory Animal Ethics Committee of Wenzhou Medical University approved all animal experiments conducted in this study (No. wydw2024‐0022).

## Supporting information



Supporting information

Supporting information

Supporting information

Supporting information

Supporting information

Supporting information

Supporting information

Supporting information

Supporting information

Supporting information

Supporting information

## Data Availability

The data utilised and/or analysed during this study are available from the corresponding author upon reasonable request.

## References

[1] Anwar WS , Abdel‐Maksoud FM , Sayed AM , Abdel‐Rahman IAM , Makboul MA , Zaher AM . Potent hepatoprotective activity of common rattan (*Calamus rotang* L.) leaf extract and its molecular mechanism. BMC Complement Med Ther. 2023;23(1):24. 36717906 10.1186/s12906-023-03853-9 PMC9885597

[2] Kisseleva T , Brenner D . Molecular and cellular mechanisms of liver fibrosis and its regression. Nat Rev Gastroenterol Hepatol. 2021;18(3):151‐166. 33128017 10.1038/s41575-020-00372-7

[3] She H , Xiong S , Hazra S , Tsukamoto H . Adipogenic transcriptional regulation of hepatic stellate cells. J Biol Chem. 2005;280(6):4959‐4967. 15537655 10.1074/jbc.M410078200

[4] Lee UE , Friedman SL . Mechanisms of hepatic fibrogenesis. Best Pract Res Clin Gastroenterol. 2011;25(2):195‐206. 21497738 10.1016/j.bpg.2011.02.005 PMC3079877

[5] Qiang G , Zhang L , Yang X , et al. Effect of valsartan on the pathological progression of hepatic fibrosis in rats with type 2 diabetes. Eur J Pharmacol. 2012;685(1‐3):156‐164. 22546234 10.1016/j.ejphar.2012.04.028

[6] Kang N , Gores GJ , Shah VH . Hepatic stellate cells: partners in crime for liver metastases? Hepatology. 2011;54(2):707‐713. 21520207 10.1002/hep.24384 PMC3145026

[7] Affò S , Bataller R . RANTES antagonism: a promising approach to treat chronic liver diseases. J Hepatol. 2011;55(4):936‐938. 21708198 10.1016/j.jhep.2011.04.023

[8] Collins PJ , McCully ML , Martínez‐Muñoz L , et al. Epithelial chemokine CXCL14 synergizes with CXCL12 via allosteric modulation of CXCR4. Faseb j. 2017;31(7):3084‐3097. 28360196 10.1096/fj.201700013R PMC5472405

[9] Liao J , Zhang Z , Yuan Q , et al. A lncRNA Gpr137b‐ps/miR‐200a‐3p/CXCL14 axis modulates hepatic stellate cell (HSC) activation. Toxicology letters. 2021;336:21‐31. 33069761 10.1016/j.toxlet.2020.10.001

[10] Li J , Gao J , Yan D , et al. Neutralization of chemokine CXCL14 (BRAK) expression reduces CCl4 induced liver injury and steatosis in mice. Eur J Pharmacol. 2011;671(1‐3):120‐127. 21978833 10.1016/j.ejphar.2011.09.174

[11] Recio PS , Mitra NJ , Shively CA , et al. Zinc cluster transcription factors frequently activate target genes using a non‐canonical half‐site binding mode. Nucleic Acids Res. 2023;51(10):5006‐5021. 37125648 10.1093/nar/gkad320 PMC10250231

[12] Saliba J , Coutaud B , Solovieva V , Lu F , Blank V . Regulation of CXCL1 chemokine and CSF3 cytokine levels in myometrial cells by the MAFF transcription factor. J Cell Mol Med. 2019;23(4):2517‐2525. 30669188 10.1111/jcmm.14136 PMC6433675

[13] Chen RX , Xia YH , Xue TC , Ye SL . Transcription factor c‐Myb promotes the invasion of hepatocellular carcinoma cells via increasing osteopontin expression. J Exp Clin Cancer Res. 2010;29(1):172. 21190594 10.1186/1756-9966-29-172 PMC3023683

[14] Winkler M , Staniczek T , Kürschner SW , et al. Endothelial GATA4 controls liver fibrosis and regeneration by preventing a pathogenic switch in angiocrine signaling. J Hepatol. 2021;74(2):380‐393. 32916216 10.1016/j.jhep.2020.08.033

[15] Bhagwat AS , Vakoc CR . Targeting transcription factors in cancer. Trends Cancer. 2015;1(1):53‐65. 26645049 10.1016/j.trecan.2015.07.001 PMC4669894

[16] Cheung KL , Zhang F , Jaganathan A , et al. Distinct roles of Brd2 and Brd4 in potentiating the transcriptional program for Th17 cell differentiation. Mol Cell. 2017;65(6):1068‐1080. 28262505 10.1016/j.molcel.2016.12.022 PMC5357147

[17] Li X , Jiang F , Hu Y . Schisandrin B promotes hepatic stellate cell ferroptosis via wnt pathway‐mediated Ly6C'° macrophages. J Agric Food Chem. 2023;71(45):17295‐17307. 10.1021/acs.jafc.3c03409 37922022

[18] Li X , Li Y , Zhang W , et al. The IGF2BP3/Notch/Jag1 pathway: a key regulator of hepatic stellate cell ferroptosis in liver fibrosis. Clin Transl Med. 2024;14(8):e1793. 39113232 10.1002/ctm2.1793 PMC11306284

[19] Lee E , Hwang I , Lee JY , et al. Hepatic stellate cell‐specific knockout of transcriptional intermediary factor 1γ aggravates liver fibrosis. J Exp Med. 2020;217(6):e20190402. 32267915 10.1084/jem.20190402 PMC7971140

[20] Piras B , Tian Y , Xu Y , Thomas NA , O'Connor DM , French BA . Systemic injection of AAV9 carrying a periostin promoter targets gene expression to a myofibroblast‐like lineage in mouse hearts after reperfused myocardial infarction. Gene Therapy. 2016;23(5):469‐478. 26926804 10.1038/gt.2016.20

[21] Wan G , Chen Z , Lei L , et al. The total polyphenolic glycoside extract of *Lamiophlomis rotata* ameliorates hepatic fibrosis through apoptosis by TGF‐β/Smad signaling pathway. Chin Med. 2023;18(1):20. 36829153 10.1186/s13020-023-00723-x PMC9951520

[22] Jinmao L , Zhang Z , Yuan Q , et al. A lncRNA Gpr137b‐ps/miR‐200a‐3p/CXCL14 axis modulates hepatic stellate cell (HSC) activation. Toxicol Lett. 2020;336(0):21‐31. 33069761 10.1016/j.toxlet.2020.10.001

[23] Shi Z , Zhang K , Chen T , et al. Transcriptional factor ATF3 promotes liver fibrosis via activating hepatic stellate cells. Cell Death Dis. 2020;11(12):1066. 33311456 10.1038/s41419-020-03271-6 PMC7734065

[24] Sahin H , Wasmuth H . Chemokines in tissue fibrosis. Biochimica et biophysica acta. 2013;1832(7):1041‐1048. 23159607 10.1016/j.bbadis.2012.11.004

[25] Berres M , Koenen RR , Rueland A , et al. Antagonism of the chemokine Ccl5 ameliorates experimental liver fibrosis in mice. J Clin Invest. 2010;120(11):4129‐4140. 20978355 10.1172/JCI41732 PMC2964968

[26] Song Z , Liu X , Zhang W , et al. Ruxolitinib suppresses liver fibrosis progression and accelerates fibrosis reversal via selectively targeting Janus kinase 1/2. J Transl Med. 2022;20(1):157. 35382859 10.1186/s12967-022-03366-y PMC8981941

