# Supplementary material for: ATF3‐mediated transactivation of CXCL14 in HSCs during liver fibrosis
Source: Clin Transl Med. 2024 Oct 2;14(10):e70040. doi: 10.1002/ctm2.70040 (PMC11446984; doi:10.1002/ctm2.70040)
Supplement: Supplementary file 1 — Supporting information [file CTM2-14-e70040-s005.doc]

# **Supplemental Information for**

**ATF3-Mediated Trans-Activation of CXCL14 in HSCs during Liver Fibrosis**

**Author-Supplied**

Xinmiao Li^1^, Lifan Lin^1^, Yifei Li^1^, Weizhi Zhang^1^, Zhichao Lang^1^, Jianjian Zheng^1, *^

^1^Zhejiang Key Laboratory of Intelligent Cancer Biomarker Discovery and Translation, The First Affiliated Hospital of Wenzhou Medical University, Wenzhou, China

***Correspondence:**

Jianjian Zheng, Zhejiang Key Laboratory of Intelligent Cancer Biomarker Discovery and Translation, The First Affiliated Hospital of Wenzhou Medical University, No.2 fuxue lane, Wenzhou, Zhejiang, P.R. China; E-mail: [zjj@wmu.edu.cn](mailto:zjj@wmu.edu.cn)

**Funding Information**

The project received support from the National Natural Science Foundation of China (No. 81873576) and the Wenzhou Municipal Science and Technology Bureau (No. Y20220023).


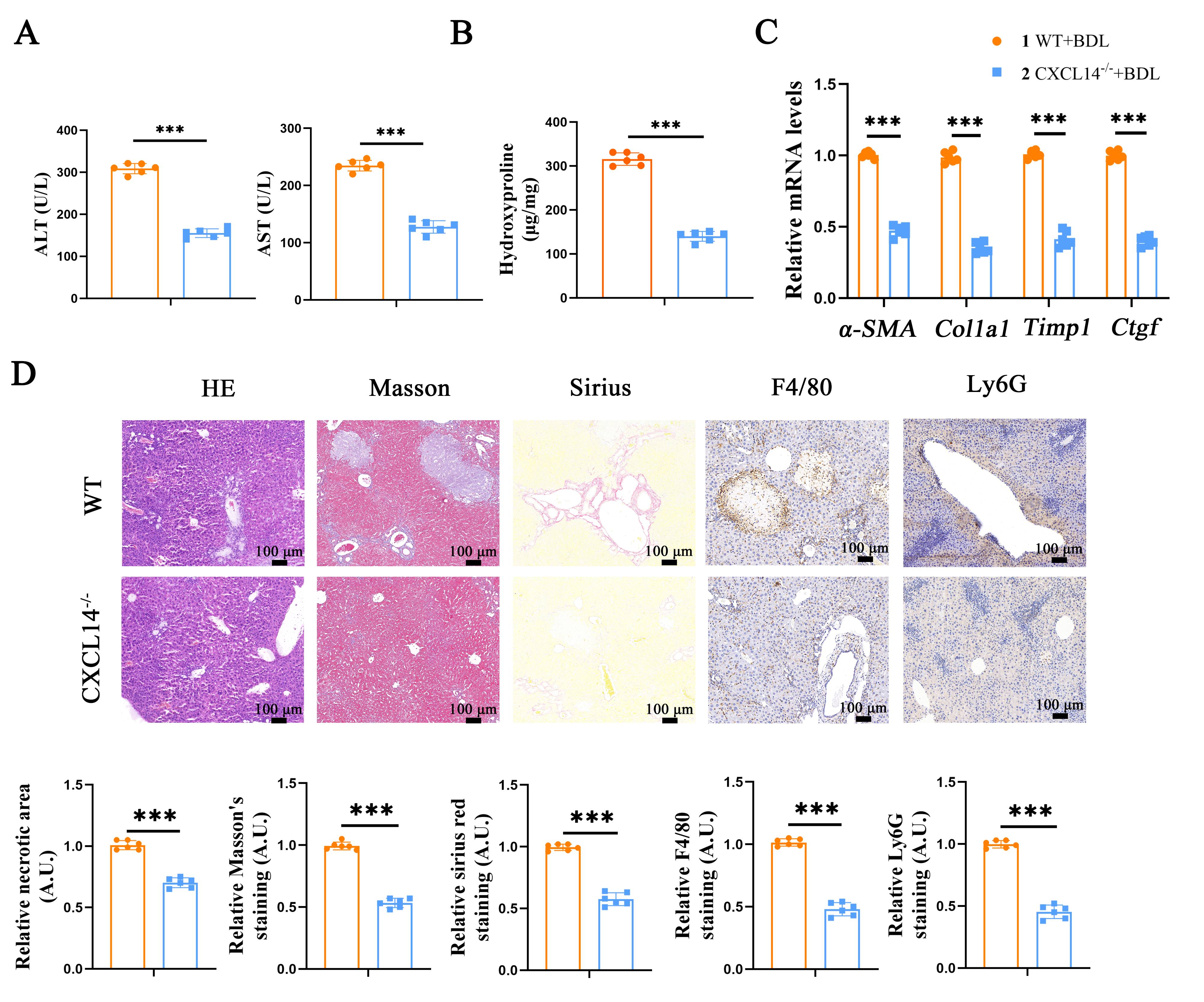
Figure S1Related to Figure 4: CXCL14 deficiency alleviates liver fibrosis in mice

WT and *CXCL14^-/-^* mice were subjected to the BDL procedure.

(A and B) Levels of ALT, AST, and Hyp. (C) mRNA levels of α-SMA, Col1a1, Timp1, and Ctgf in the liver tissues. (D) HE, Masson, Sirius, F4/80, and Ly6G staining. Each value is the mean±SD of six experiments. ****P* < 0.001.


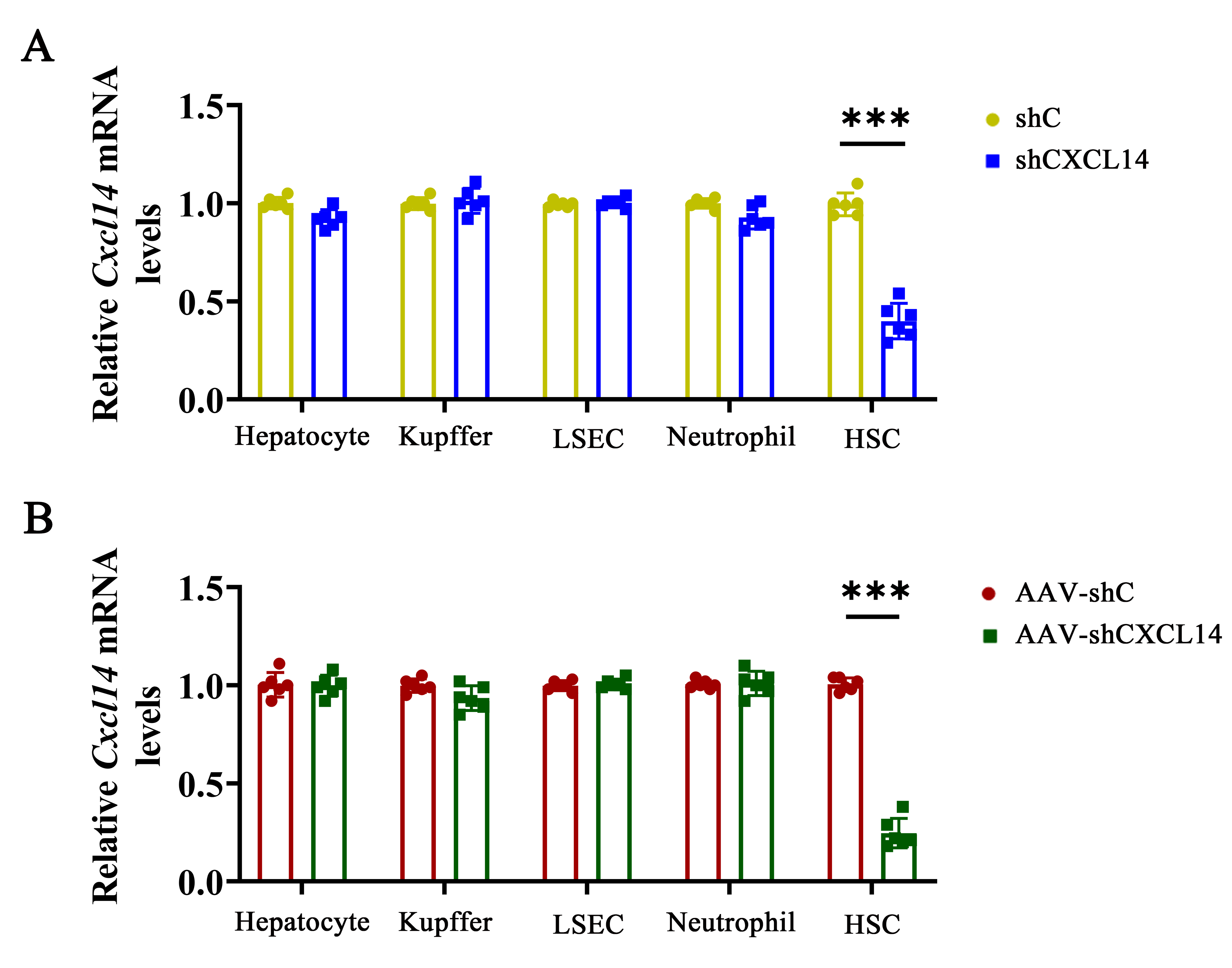
Figure S2 Related to Figure 5: HSC-specific CXCL14 deletion mitigates liver fibrosis in mice

The mice were injected with Lenti-shC, Lenti-shCXCL14, AAV-shC or AAV-shCXCL14 and then isolated the primary HSCs, hepatocytes, KCs, LSEC, and neutrophils to detect the mRNA levels of CXCL14.

(A and B) mRNA levels of CXCL14 in the primary HSCs, hepatocytes, KCs, LSEC, and neutrophils. Each value is the mean±SD of six experiments. ****P* < 0.001.


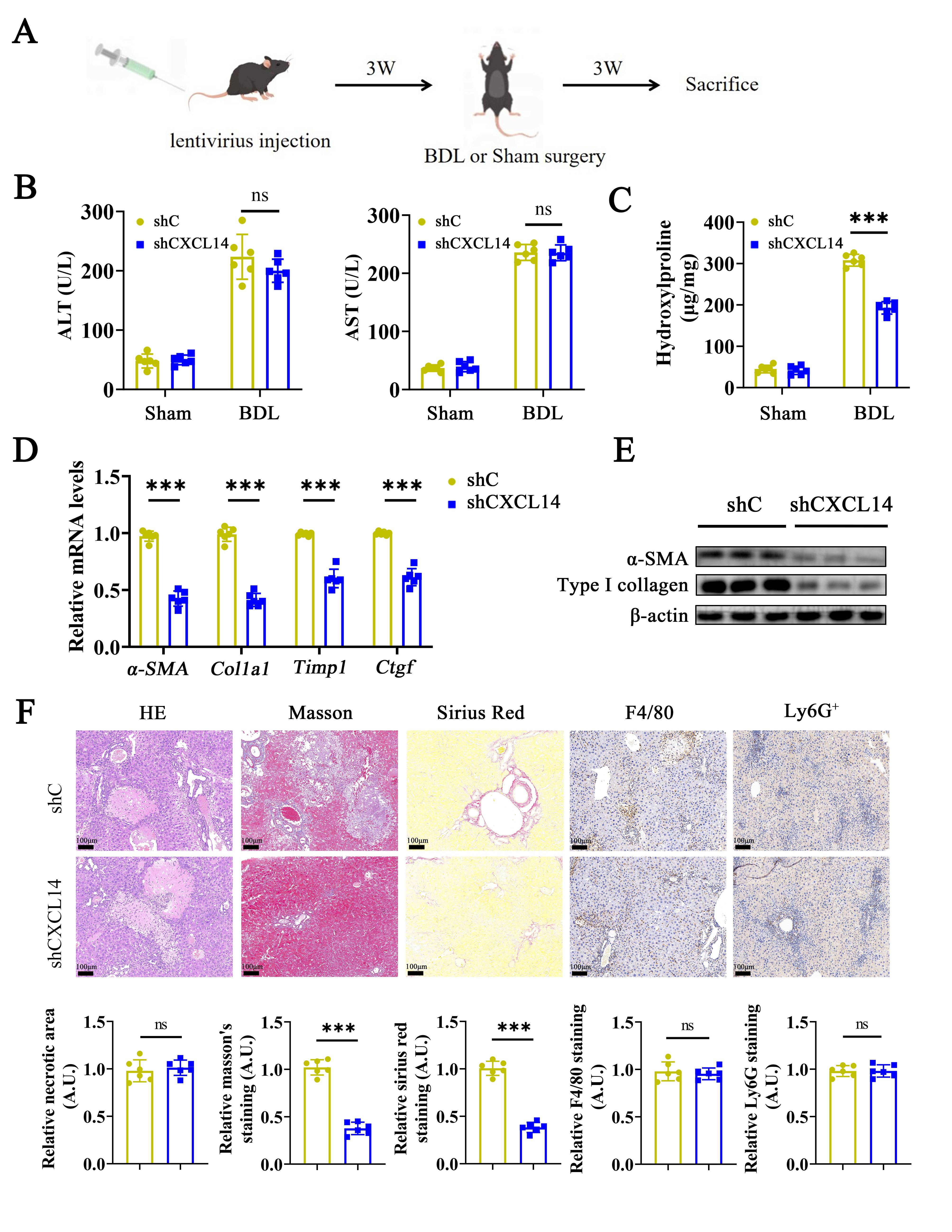


Figure S3 Related to Figure 5: HSC-specific CXCL14 deletion mitigates liver fibrosis in mice

Lenti-shCXCL14 or Lenti-shC packaged lentiviruses were injected into C57/BL6 mice and then subjected to the BDL procedure.

(A) Animal experiment design. (B and C) Levels of ALT, AST and Hyp. (D) mRNA levels of α-SMA, Col1a1, Timp1, and Ctgf in the liver tissues. (E) Protein levels of α-SMA and type I collagen in the liver tissues. (F) HE, Masson, Sirius Red, F4/80, and Ly6G staining. Each value is the mean±SD of six experiments. ****P* < 0.001. ns, no significance.


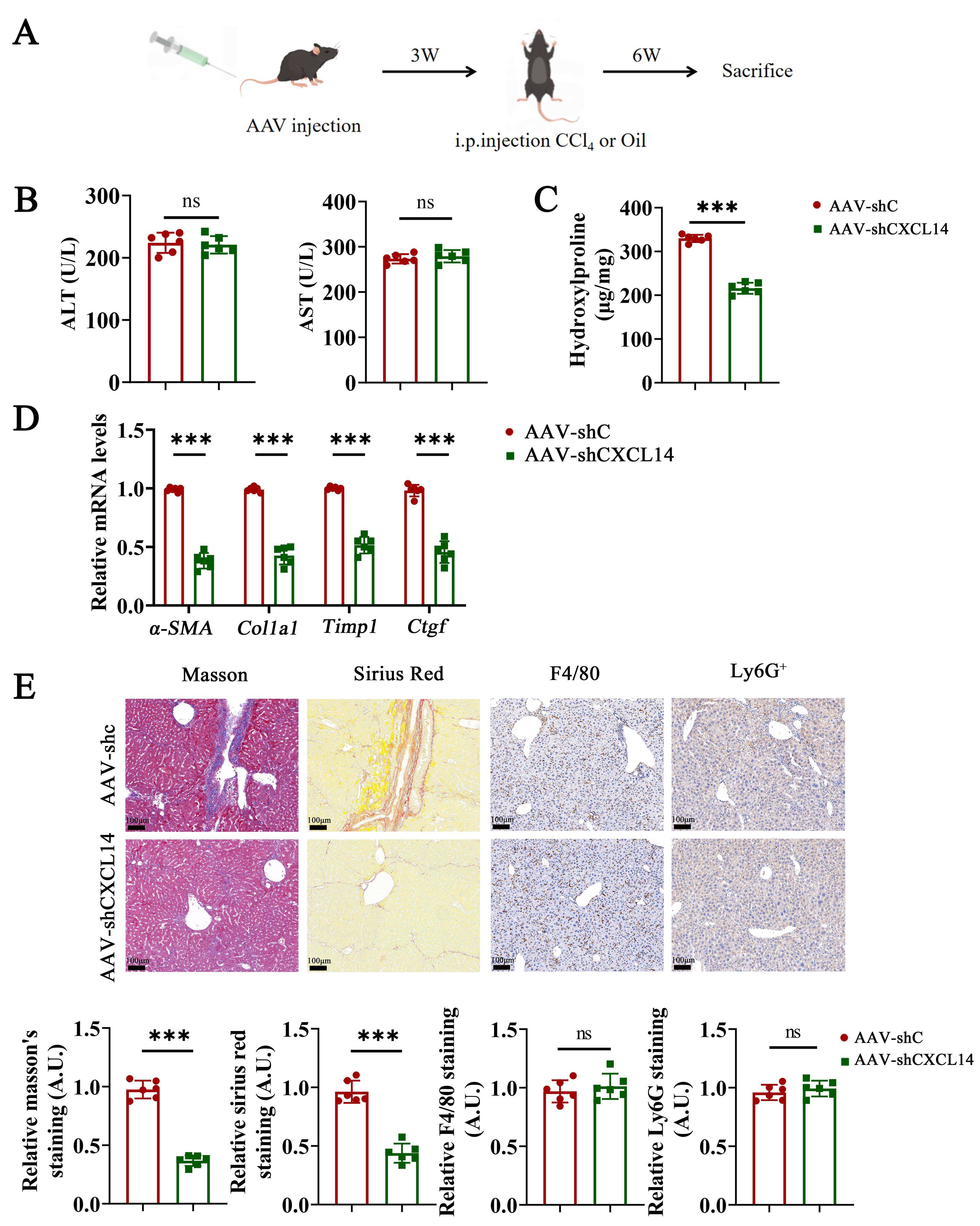


Figure S4 Related to Figure 5: HSC-specific CXCL14 deletion mitigates liver fibrosis in mice

AAV-shCXCL14 or AAV-shC were injected into C57/BL6 mice and then subjected to CCl_4_ injection.

1. Animal Experiment Design. (B and C) Levels of ALT, AST and Hyp. (D) mRNA expressions of α-SMA, Col1a1, Timp1, and Ctgf in the liver tissues. (E) Masson, Sirius Red, F4/80, and Ly6G staining. Each value is the mean±SD of six experiments. ****P* < 0.001. ns, no significance.


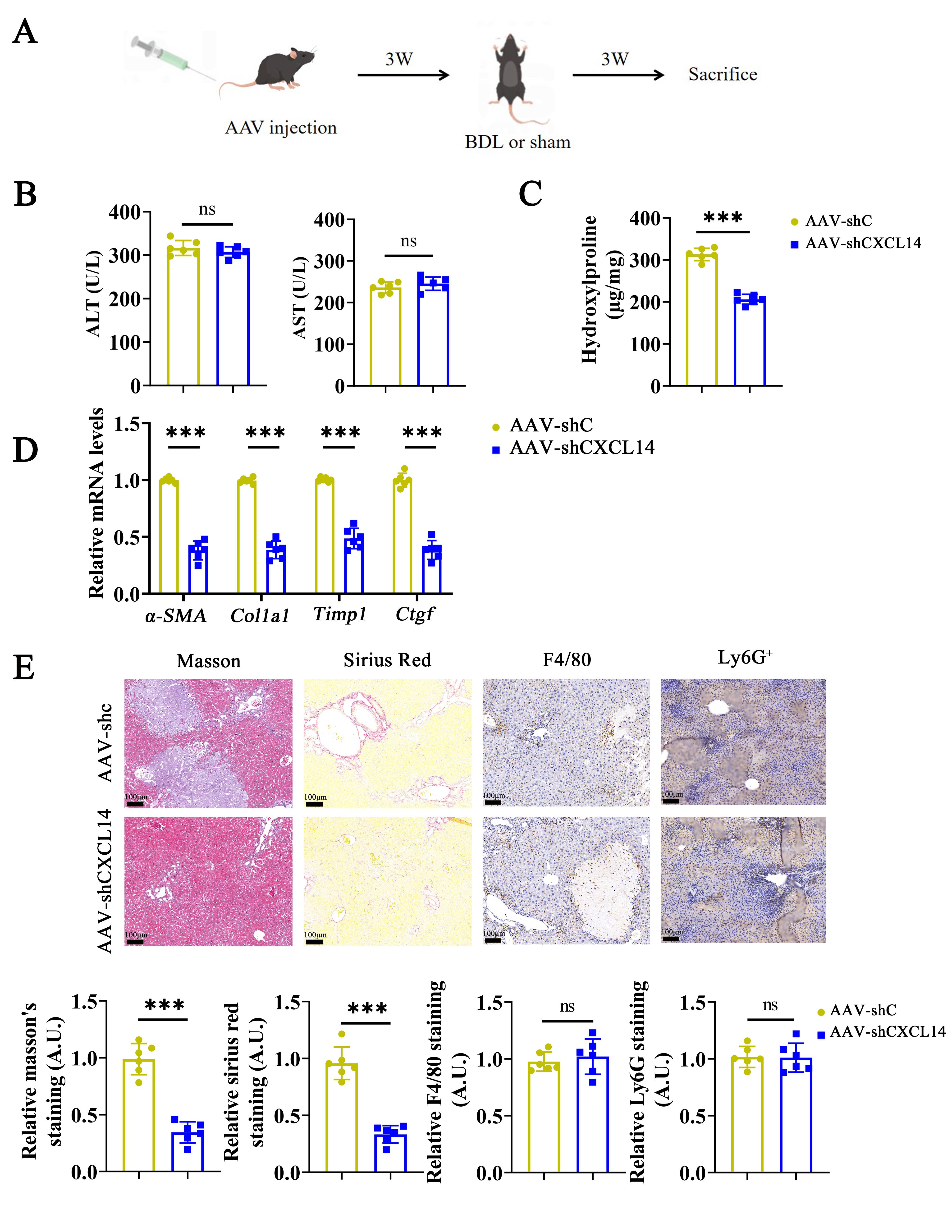


Figure S5 Related to Figure 5: HSC-specific CXCL14 deletion mitigates liver fibrosis in mice

AAV-shCXCL14 or AAV-shC were injected into C57/BL6 mice and then subjected to the BDL procedure.

1. Animal experiment design. (B and C) Levels of ALT, AST and Hyp. (D) mRNA expressions of α-SMA, Col1a1, Timp1, and Ctgf in the liver tissues. (E) Masson, Sirius Red, F4/80, and Ly6G staining. Each value is the mean±SD of six experiments. ****P* < 0.001. ns, no significance.

**
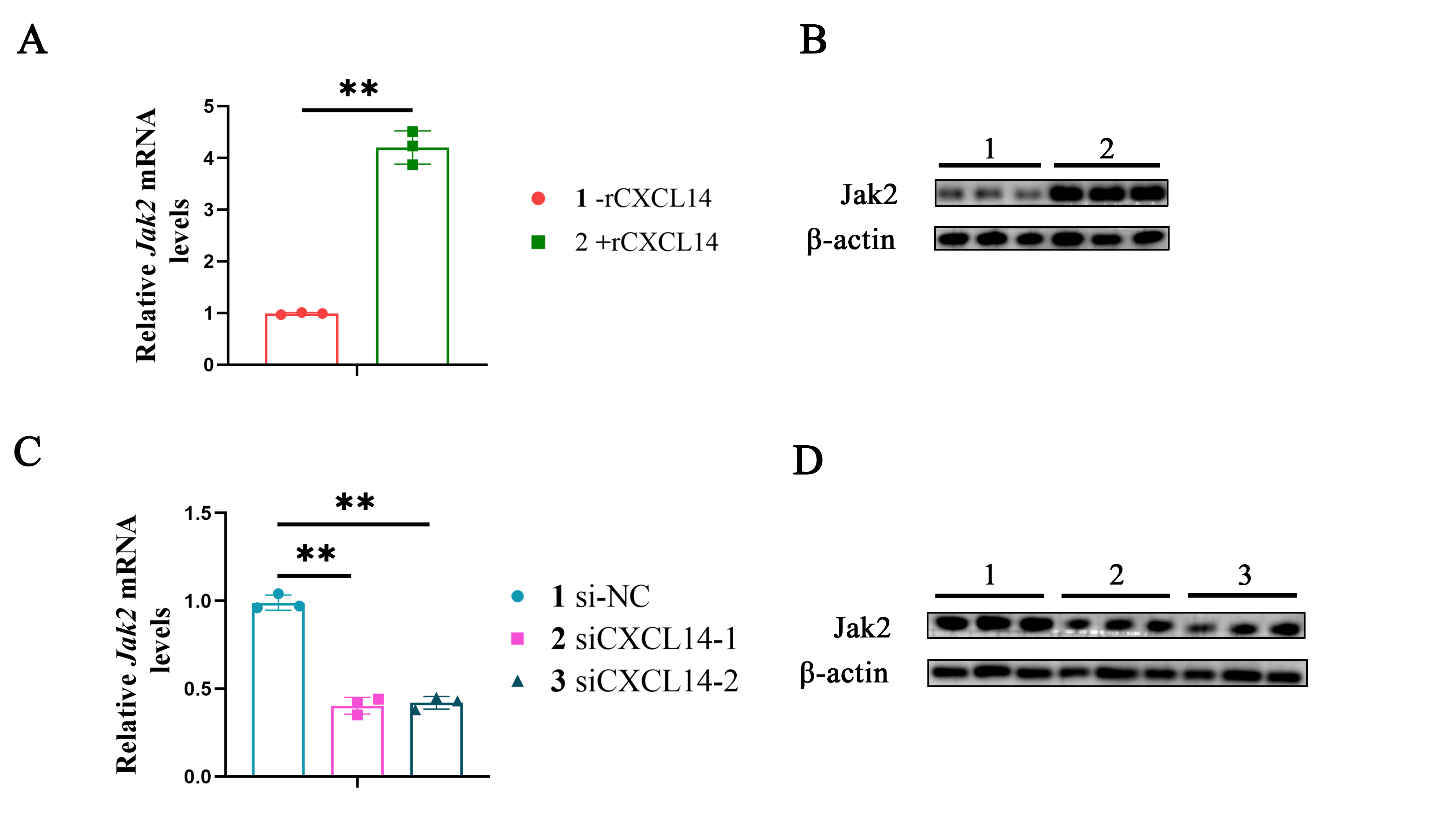
**

Figure S6 Related to Figure 6: Jak2 is involved in the role of CXCL14 in HSC activation

(A and B) mRNA and protein level of Jak2 in LX-2 cells. (C and D) mRNA and protein of Jak2 in LX-2 cells. Each value is the mean±SD of three experiments. ***P* < 0.01.


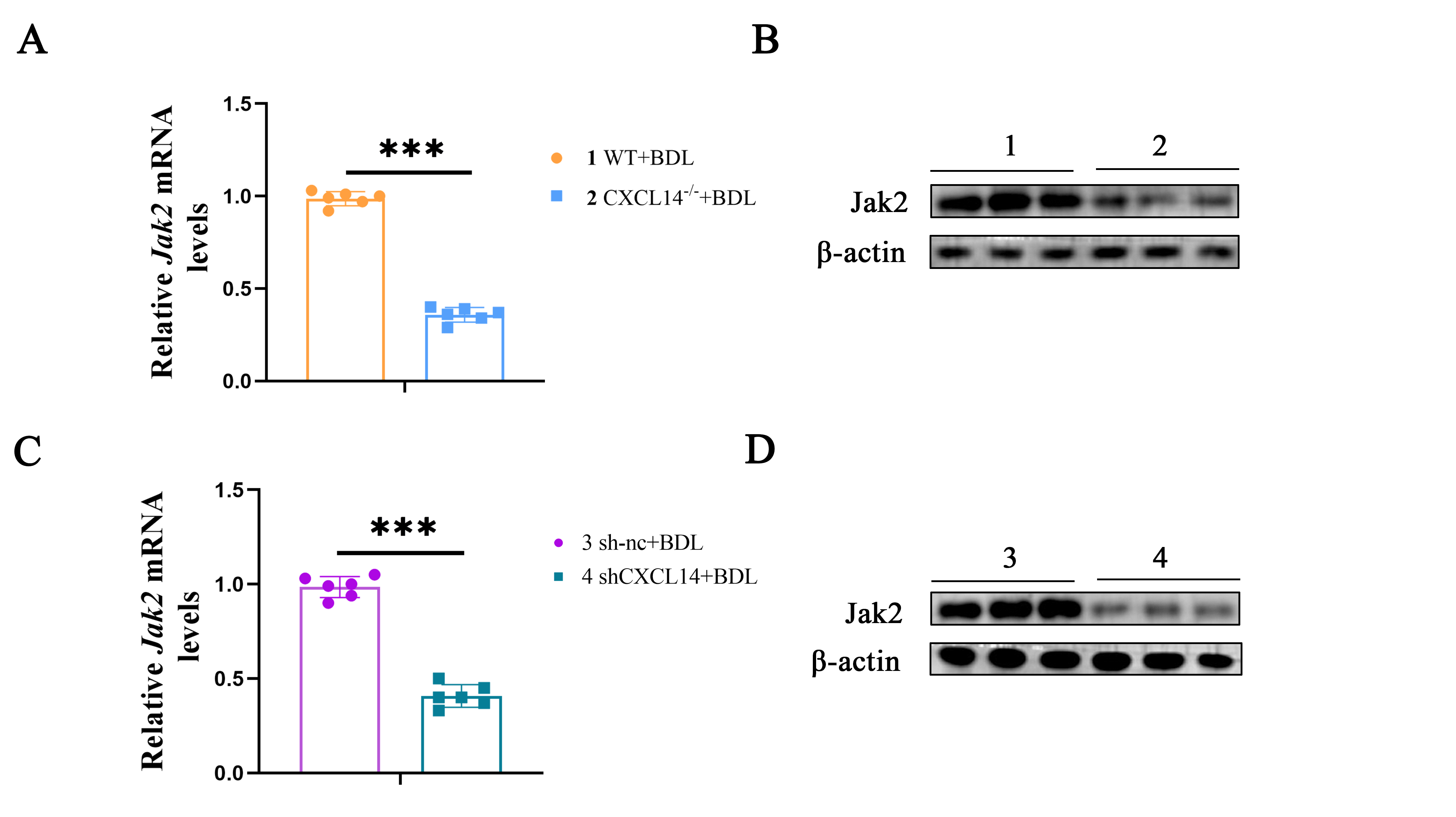
Figure S7 Related to Figure 6: Jak2 is involved in the role of CXCL14 in HSC activation

(A and B) mRNA and protein level of Jak2 in liver tissues isolated from the WT and *CXCL14^-/-^* mice subjected to the BDL procedure. (C and D) The mice were injected with AAV-shCXCL14 or AAV-shC and then subjected to the BDL procedure and then isolated the liver tissues to detect the mRNA and protein level of Jak2. Each value is the mean±SD of six experiments. ****P* < 0.001.


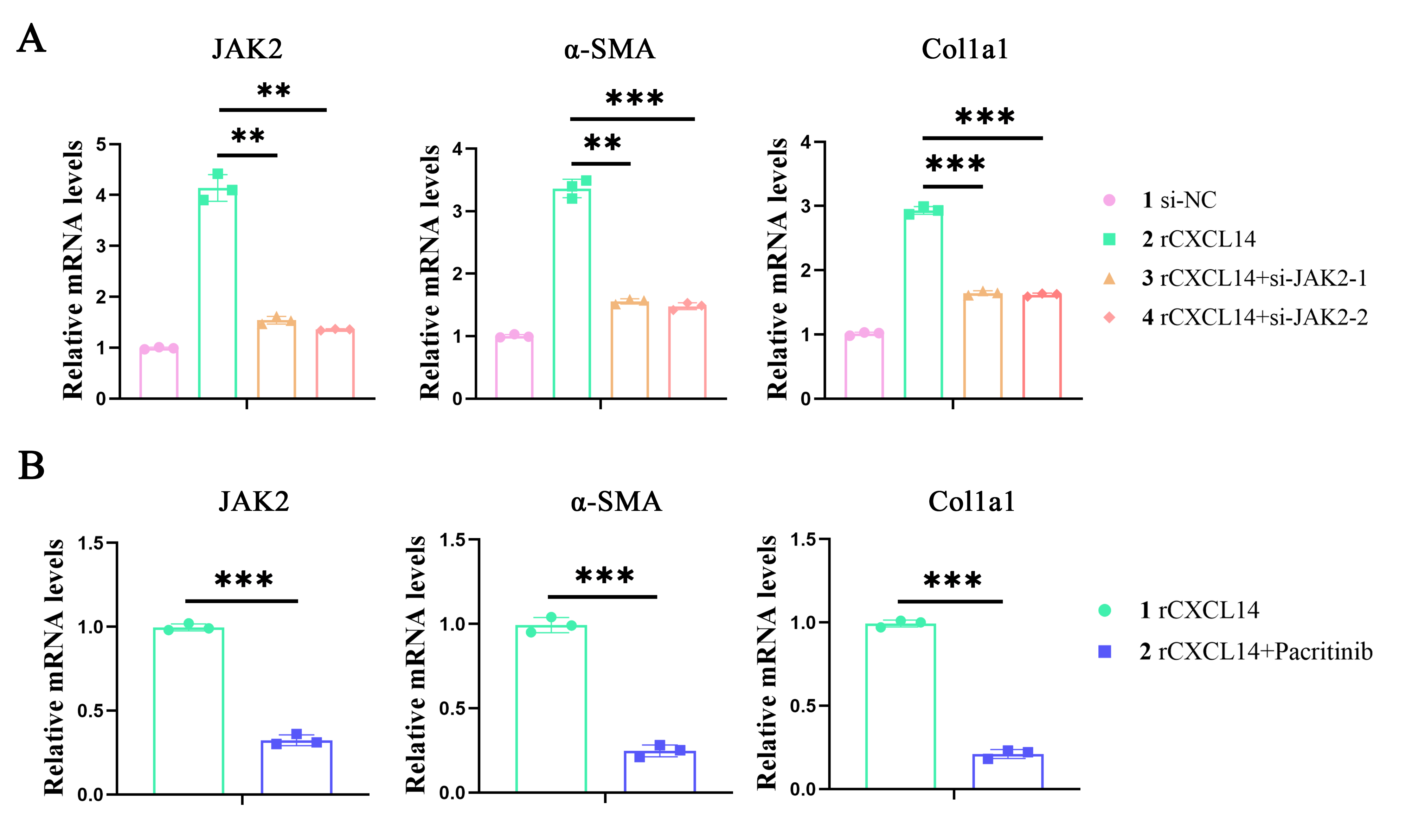
Figure S8 Related to Figure 7: CXCL14 promotes HSC activation as well as liver fibrosis via Jak2

(A and B) mRNA expressions of Jak2, α-SMA, and Col1a1 in LX-2 cells. Each value is the mean±SD of three experiments. ***P* < 0.01, ****P* < 0.001.


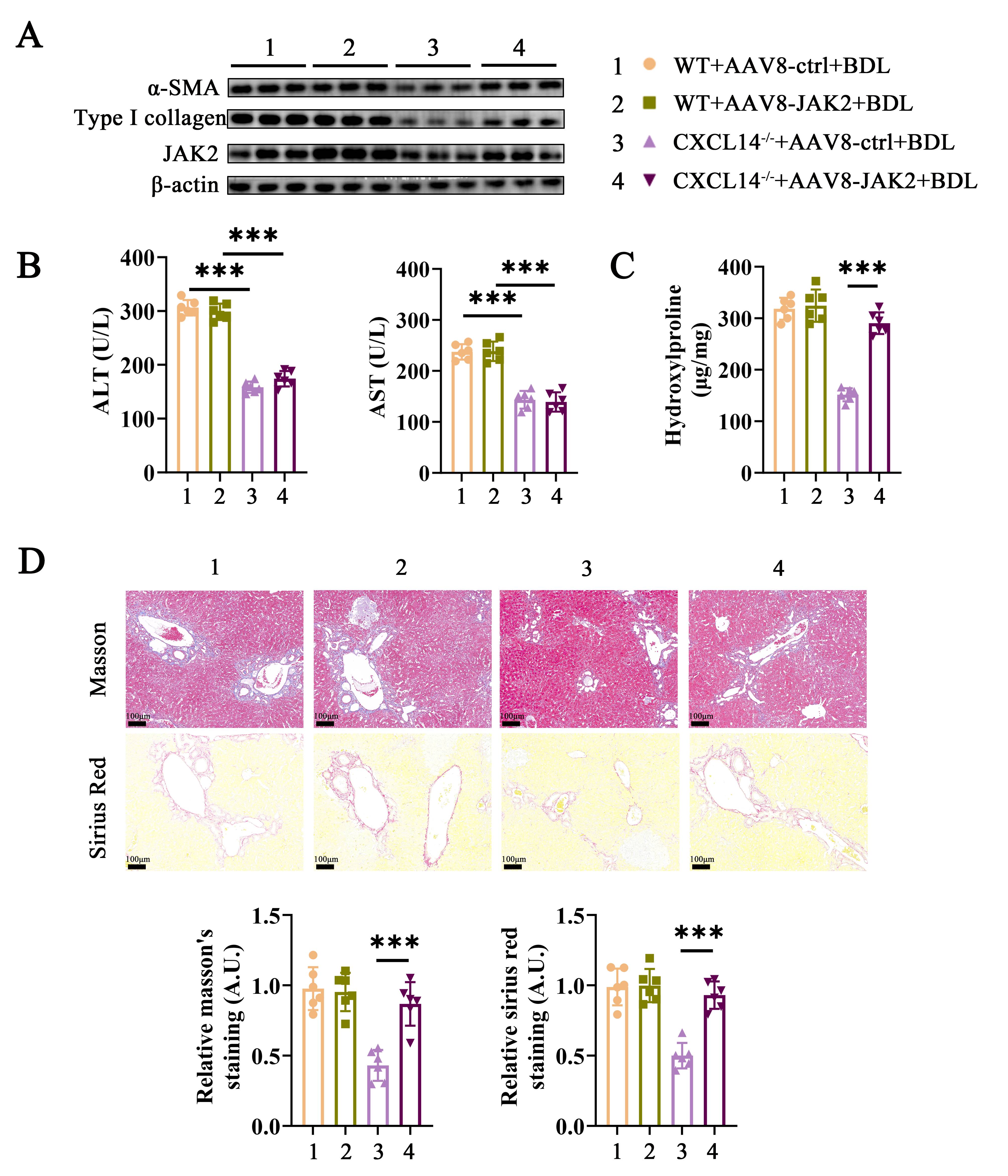


Figure S9 Related to Figure 7:CXCL14 promotes HSC activation as well as liver fibrosis via Jak2

AAV8-JAK2 or AAV8-ctrl were injected into WT and *CXCL14^-/-^* mice and then subjected to BDL procedure.

(A) Protein levels of α-SMA, type I collagen, and Jak2 in liver tissues. (B and C) Levels of ALT, AST and Hyp. (D) Masson and Sirius Red staining. Each value is the mean±SD of six experiments. ****P* < 0.001.


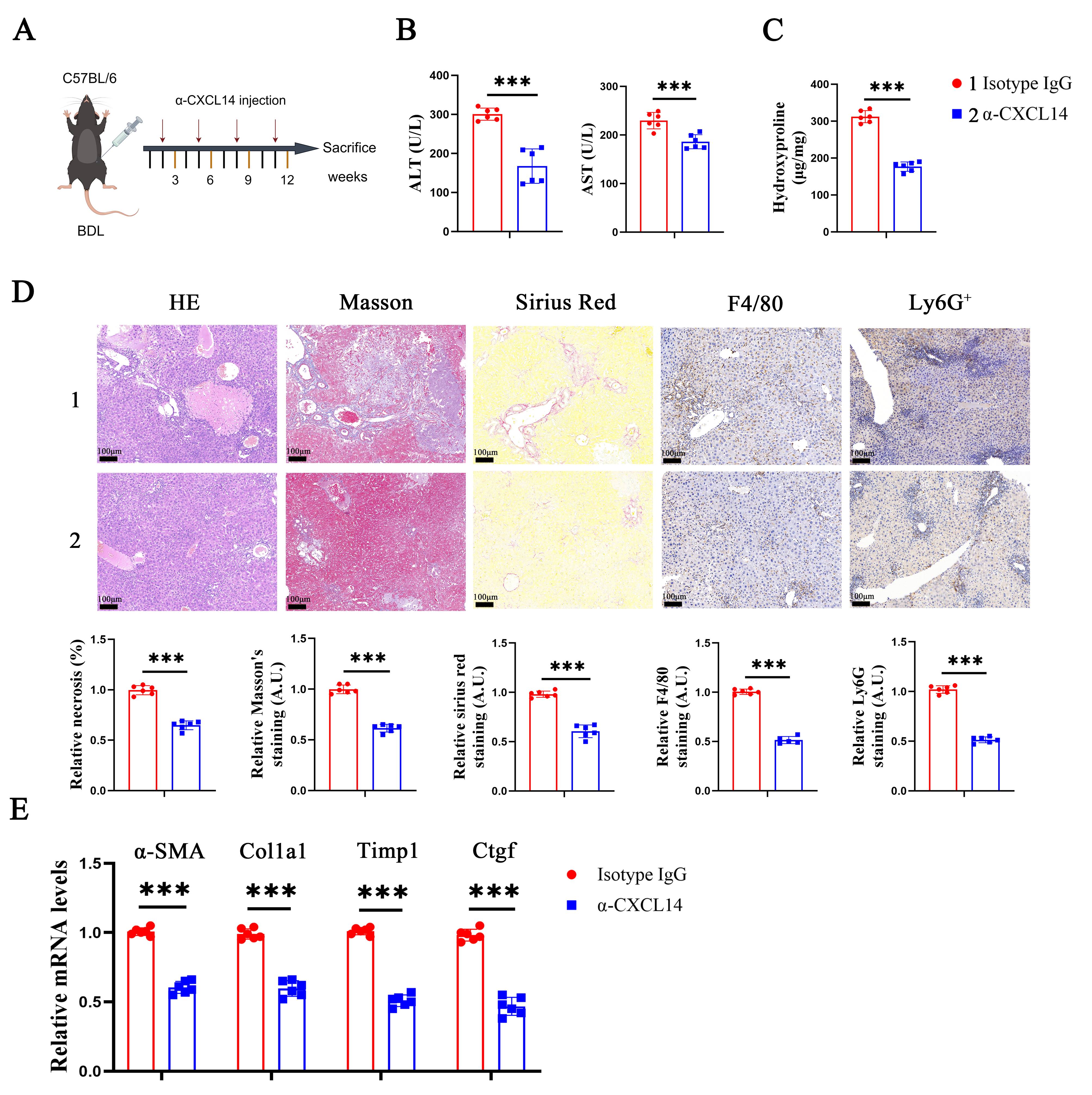
Figure S10 Related to Figure 8: Inhibition of CXCL14 ameliorates liver fibrosis *in vivo*

Mice with BDL-induced liver fibrosis were injected with α-CXCL14 (15mg/kg) or an isotype IgG (control) every three weeks for 12 weeks.

(A) Animal experiment design. (B and C) Levels of ALT, AST and Hyp. (D) HE, Masson, Sirius Red, F4/80, and Ly6G staining. (E) mRNA levels of α-SMA, Col1a1, Timp1 and Ctgf in the liver tissues. Each value is the mean±SD of six experiments. ****P* < 0.001.

# Table S1

# **1.**The primers used for qRT-PCR (mouse)

| Primer | Sense | Antisense |
| --- | --- | --- |
| CXCL14 | 5'-GTAAGTGTTCCCGGAAGGGG-3' | 5'-CCTCGCAGTGTGGGTACTTT-3' |
| Jak2 | 5'-TATGATGAGCAAGCTTTCTCACAAG-3' | 5'-TCCAAATTTACAAACTCCTGAACC-3' |
| α-SMA | 5'-GCCATCTTTCATTGGGATGGA-3' | 5'-CCCCTGACAGGACGTTGTTA-3' |
| Col1A1 | 5'-GCTCCTCTTAGGGGCCACT-3' | 5'-CCACGTCTCACCATTGGGG-3' |
| TIMP1 | 5'-GCTAAAAGGATTCAAGGC-3' | 5'-GCACAAGCCTAGATTCCG-3' |
| Ctgf | 5'-CTTCTGCGATTTCGGCTCC-3' | 5'-TACACCGACCCACCGAAGA-3' |
| β-actin | 5'-GGCTGTATTCCCCTCCATCG-3' | 5'-CCAGTTGGTAACAATGCCATGT-3' |

# **2.**The primers used for qRT-PCR (human)

| Primer | Sense | Antisense |
| --- | --- | --- |
| CXCL14 | 5′-TCCGGTCAGCATGAGGCTCC-3′ | 5′-CACCCTATTCTTCGTAGACC-3′ |
| ATF3 | 5′-ATCTCCTTCACCGTGGCTAC-3′ | 5′-AGGACCTGCCATCATACTGC-3′ |
| Jak2 | 5'-GAAGAACTTCAGCAGTCTTAAAGATC-3' | 5'-CCATGCCAACTGTTTAGCAACTTC-3' |
| α-SMA | 5'-GTGTTGCCCCTGAAGAGCAT-3' | 5'-GCTGGGACATTGAAAGTCTCA-3' |
| Col1A1 | 5'-CCCGGGTTTCAGAGACAACTTC-3' | 5'-TCCACATGCTTTATTCCAGCAATC-3' |
| β-actin | 5'-GGCACCCAGCACAATGAAG-3' | 5'-CCGATCCACACGGAGTACTTG-3' |

**Table S2**

# **1.**Antibodies for Western blot

| Antibodies | Source | Identifier |
| --- | --- | --- |
| α-SMA (mouse) | abcam | ab124964 |
| Type I clollagen (mouse) | abcam | ab270993 |
| JAK2 (mouse) | abcam | ab108596 |
| JAK2 (human) | abcam | ab108596 |
| β-actin (mouse) | abcam | ab8226 |
| β-actin (human) | abcam | ab8226 |

**2.Antibody for Immunohistochemical staining.**

| Antibodies | Source | Identifier |
| --- | --- | --- |
| F4/80 (mouse) | abcam | ab300421 |
| Ly6G (mouse) | abcam | Ab238132 |

1. **Antibodies for Immunofluorescence Staining**

| Antibodies | Source | Identifier |
| --- | --- | --- |
| CXCL14 (human) | abcam | ab264467 |
| α-SMA (human) | abcam | ab124964 |
| α-SMA (mouse) | abcam | ab124964 |
| Type I clollagen (human) | abcam | ab138492 |
| Type I clollagen (mouse) | abcam | ab270993 |

The sequence of mouse Lrat promoter:

1 CCTTAA AGAGAG GCATCC GGGGTC TGACTG AGAGTG CCAATC CCGTCT TGGTGA CTCCAA

61 GGCACT GATATT TTCTCA GATCTT GGATGT CAGGAA ATATCA ACATCT ACAGCT AACACA

121 CATTCA AAAGGA CATGCA AGCACT CCTGTG GAATTC CTGACA AAAAGT ACTACC TGAAGG

181 TCTGCA TGAATA AACATC AAACCC ACACTG ATGTGT GTTTGA AAAATC AAGTTT TGTTTT

241 GCTCCG GGAGAA AATACC ATGACA AAGGCA GAGGAC ATCAGC TATGTA ACAATG GATGGG

301 CAAGGC GCCATT CAGATT GTTTCC TGTAAT GGCCAT TACTAC AACAAT CAGGAA ATTCTG

361 AGGAAG CCTGAA GTTTAC ATGATA ATACTG CTGATA GTTCTT TGACTT TGATAA TTATAA

421 AAGTGT GATTAT ATACAG AGCAGG GTTGAG GGACAA GTGGAC ATCAAA GCTACA ACTTGC

481 TAACTG GTTGGA AAAATG TGTGTG CACACA TGTGTC CATATA TACAGG TGAAGG GTAAAG

541 TGTGGG AATTCT TTGGAC ATTTTT TTTTCA GATATT TAAGAG ATCTAT TATGAA AATTAA

601 GGACTA AAAAAG TGTCTT CCTTTC TCTATT GATTGG GTCCTT TCCATT TTAGGG TTGAAC

661 ATCTTG AGTGAG TTCAGC TTTGAC ATTCAC TTCATG TGCTTT GTTTCT GAGTTA AGTATT

721 TAGATG CTACAC TGGCCA GAATAT TGGAAA AATGGT CTATCT AGAGAT CCTCAG TGGCCT

781 CTTCTA GCAAGT CTCAGA AGGAGA CAGCCC ATAGCT AATATA CTTTAA GAAACA GAGAGG

841 GCAACA GAAACC AAAGAA TTGCAC ACCCAT CTAACA CACACA AGAGCT GATATC AGCATC

901 TAGACT TATAAT CAGCCC AAATAC ACATGT CTATTC GCCTGG ACTTTG TGTAGA ATCAAA

961 ATAACA GCCAGG ACATTA TATCTC TATTAG AGCAAA ACAATC CTTCTA CAGTGG ACCCTG

1021 AAAAAT GCAACT TAGCCA AAGCCC ATGCCA GGGACG TCAAAA TAGCTG TAATAA ATATGC

1081 TATATG TATTTA AAGATG ATATGA ATAAAT CCATTA ATTTAT GGAACA CAAAGC ATAGAA

1141 TGAAAT TTAAAA AATGTT CAAGAC ATGGCG GTGAAA AAAGAA TCAATG AAGAAA CCCCAA

1201 ACTGAT GGAAAA CAAAAT GAAAAC TGTAGG AAGTCA AACGGG ATCCTC AGAGGC GAGTCT

1261 CAGTAA CAGAAG ACAAGA GAAGGA AGAGAG AATCTT ACACAG TGAAGA TAAGAT AAAAGA

1321 AATGCT GATACC TCAGTC AAAGAA AATCTG AGCTCT GGAGCA GACCCA ATTTAA GAATAA

1381 TAGGAA CAAAGG ATGCAT GAGAAA CTCAGG TCAAAG GTACTA GGTATC TACATA GAAATC

1441 AAAAAC AAGCAA ACAAGC AGACGT CTTATT AGGAAC TTAATA GAACTG AAAGCT CTAGGG

1501 TAAAAA GAAATA GCACCC AAAAGG AGAAGG TGGCAA TAAATA ATCAAG CTCAGG GTTGAA

1561 ATCAGT AAAATA GAAACA AAATAT TTACAA AGAATC AATAGG ACAAAG AACTGG TTCTTT

1621 GAGAAA ATCAGT TAAGAC TGACAA ACCCTT AGCCAA TTGACT AAGAGA CAGCCT AAGCAG

1681 ACCCAC ACTAGC AATCCC TGTACC CAGGAG TAGGAC CAACAC AAAACC AACTCA ATGCAG

1741 TTTTGG ACGTAC TTTATC TCAATG TTTTGT GACAGC ATTTTT TTTAAC TTTACA AATCTT

1801 TGAGGA AATATT ACATAG TTTGTG TTTTTA TAGGAT TCCTGT ATATAA AAGGTA CATGTC

1861 TCTATA TTTATA TATGTG TCTTGT GCTTTT TATTTG ACTCTT TTTCTT CTGCTT TCTAAG

1921 GAAGAC AGTAGG GTGTGG ATCTTA GTAGAA GGGTAG ATGGGG AGGAGC TTGTTA GGGGAG

1981 GGGAAC TGAAAT CAGAAT GTATGA AATCAG TATTGT ATGAAA AATTTG TTTTCA ATAAAA

2041 ACAATG AAATAT ATATTG AAAAAT TTGTTT TCAATA TAAGCA TGTTTA TATTTC CATTTA

2101 GTGCTC CATCAT GAGGCT GAGTGT GGGCTT CCTTGA AGATAA GACTAA ATAGTC CTTTTA

2161 GTTGGC AAAATT TGCAAT TCAGGT TTTGTC TCTTAA GGTGAT TGATGA ATTAGC TGCTCA

2221 GTATTT AATATT ACATGG GTTTAA ATGATA CATAGA AATCTA CTTTTA ATTATA TTTTAA

2281 TCTATA GTTAAG TATCAC AATAAA ACAGTG ATTTTG TATAAT AATTTT TGTTTA AAACTT

2341 TGCTGG TCATGG TGCTAT ATGCCT TTAATG TTAGCA TCTGAG AGTCTG AAGCAA GCAGAT

2401 CTCTGT GGCTTC AAGGCC AGGGCT ACAAGT GAGGCC CTGTCT CTAAGT AAATAA ATACAT

2461 TTTGAC TTGGGG TCCAAC TTTTCT ATATTT TTTTAT CATTAA TATGAT TCTCCC ACTCAC

2521 ACCTCA CCATCA AACTCA TTTAGT CCTAAA GACTGT CAACCA ACAACG AAAGAG CTTATA

2581 AAGGCC GTTCTA ATAGAT GTGGAC AAGTGC TCTCAA GACATG GCTCAG GGGTCT CTCACT

2641 TTTCTG TTAGCA TGGCGA TCAGGC CTCTGC ACCCTA GTGGAT GGCACC ACTCAG CAGGTT

2701 TGGTAG CTTCTT CAGAAG CTGCAG GACAAG ATGGGG GTGGGT ATCTTC TGTTTT GTTTTT

2761 CTAGTA CTAGGG ACAGAA CCCAGA TCCTCA CACATG TGCTCC AGTGAG CTACAC GTCCAG

2821 TTCCAG ACTCTT TCCACC CACAGC CTTACA CGTTGT ATCTAC TCCCTC TGTTCC TGGCAG

2881 GGGAAC AAAACC CTGCCT GCCCTT AGCCGT GATTCT ACCTTG GTCTAT GGTTTC GAACCC

2941 ACTGCT TCCCAC TGGTTT TCTTTC TTTTTT CCTATT TCTGAG ACAGGG CCTCAA CATGTC

3001 GTCCTG GCTGAC CTGGGT CTCAGG ATGTGG CCTCTC ACTCAC AGAGAT CTGCCT GCCTCT

3061 GCTTTC TGAGTG CCGGGA TTAAAG ACACAA GTCACC ATGCCC AGCACT TAAGGT GTTTTT

3121 CACTGT CGTGTG GCTTTT GTCTCC CCATCT CACCCC CTTTAT TGTTCA TAAAAC CGCGTA

3181 GAATCC CCACTT AAAATA ACTTCC ATTCGA GCCTAT TTCTTT TTTGCA AAGTTT CTTGAA

3241 ATAGTA TAAACT TTTATT ACTGTG TTTGCA TGTGAG GGCCCG TAGCAC CCAAGT GGAGCT

3301 CAGAGA ACCTCC TGCCGG TGGCTC TCGCCT ACCATG GACTCT TCAGAT GGAACT TGGGCG

3361 GCTTCC TAATAA TCTGTG TACCTC TCCTAG CCCTTC CTCCCT ATACCA GTAAGA ACAATG

3421 TGTTGA AAGAGG ACCACT GGCCTT AAGACA AGTAGA AAAAGC ACTACA AGTGAC AATGGT

3481 GTGGGA AAGAAG TACTGG TTGATT TTGTTG AACTTC CAGGAA GGGGAC AGTGCA TGGCAG

3541 AGCAGG TAGACG GTAAGG AAAAAG ATGATT GTGGAT GGGTTG ATGAAA GATCAT GCCTAC

3601 TTCTTC TTTCTA TCCATT TGAGGC AGTGGG GAGATG TGGGGT GTGCTG CAGTGA GATTTA

3661 CAATCA TCTGTG AAGTGA ACGAAT GACCAA AAGTGC AGCGAC ATGGAC AGATTG CTTATA

3721 GCAGTC AGGGGG CAAAGG CTCAGG CCTCCA AAGAGG GGTGGC CTCAGG CCTCAG CCTGGT

3781 CTAGGT TCCATC ATGGCA GAGATC TCAGCC GGGCAG TGGTGA TGCATG CCTGTA ATCCCA

3841 GCACTT GGGAGG CAGAGG CAGGTG GATTTC TGAGTT CGAGGC CAGCCT GGTCTA CAGAGT

3901 GAGTTC CAGGAC AGCCAG GACTGC AGAGAA ACCCTG TCTCAA AGAGAG AGGAGA GGAGAG

3961 AGAGAG AGAGAG GAGAGG GAGAGA GAGGGA GAGAGA GAGAGA GAGAGA GAGAGA GAGAGA

4021 GAGAGA GAGAGA GAGAGA GAAAAG AGATAG GAGAGA GAGAGA GAGAGA AAAGAG ATAGGG

4081 GAGAGA GAGAAG AGATAG GAGAGA GAGAGA GAGAAA AGAGAT AGGAGA GAGAGA GAGAGA

4141 AAAGAG ATAGGG GAGAGA GAGAGA GAAGAG ATAGGA GAGAGA GAGAGA GAGAGA GAGAGA

4201 GAGAGA GAGAGA GAGAGA GAGAGA GAAGAG ATAGGA GAGAGA GAGAGA GAGAGA GAGAGA

4261 GAGAGA GAGAGA GAGAGA GAGAGA GAGAGA GAGAGA GAAAAG ATAGCC TGGAGC TTAAGC

4321 TTTCTT AGTTTT GTGGCG CTTGTG GCCTTT GTCTGG GGGAAT TTACTC ATTTAC ATAATG

4381 AATTAG CACAGA AATTCA GGCGGG TCTGAG CACCCA AAGCCC AGCCCT GCAGAG CCCCAG

4441 CAGCCT CCTTAG GCAGCT CCCCCA CCCCCT TCGACT GTGCCT GGGTTC TGCTGC AAGCGT

4501 GCACCG CCTTGC AGAACC ATAACG TTCTCT TTCCTT AAGTCT CCCTGG ACGGCA GGGAGC

4561 TCTTTA CCAAAA GCTACC AGTCGT TGTGTG AAGCGA AGCTGG GACCTG ACATCA AAGAAA

4621 CTTGGC TTGACT CCTGCT TTGCTG GAGGGC GTTGGG GTTTTT TTGATG ACCGTT GGAAGG

4681 CGCGCG CTCAAC AGGCAC TGGAGC CTTGGA GCGTCC AGACTG TTGGGT TCCGAT CCTCCG

4741 CGCTGG GGCACT GGACGA ACAGAG AGCCAT TCCACT TCTCCG CGTGCC ACCTGA GAGCTC

4801 ATGGAT GCTTGC GTGTTT AAAACG ACAAAG ATCAGT TTAAGT AGGATC TGCAGG AATTCG

4861 CGGAGC CGGCAC TTTGGC GAATCA AAGCAA ATTCTT TAGTAA GCTGAG GGCCAC GGTATT

4921 ATTATC GGTTAG AGCGGG TCCCAT ACCGAA ATTTTA CAGAAG GCGCTT CTCACC GCCAAG

4981 AAAGGT GCTCCA TTCCTG GCAGGT GCAGGA TCTTTC GCGCGG CCACGC CTCTGG CATCTC

5041 TCCTAC GCTGGC TGTTTA AAGGTC TCCCTA GTGAAG CAGAGC GCTCGA GGAGAA GTCAGG

5101 GTGCAG AACGCC CGTGCC CAATCC GTGTTC TCCGGC CTGTGA CCGCAT TCGGCC CGCCCT

5161 CTGCAC CTCTAT AAGAGC CACAGC GAAGAG CGCACC CAGAGC ACCGCC CCCAGG CACACT

5221 ACCTCT TCAGCT CCTCAA GCGCGG GTGCCA CTGTTC CCACAG CCGACC GACACT ACTTCC

5281 TTTCTT TGACCC CCTGCA CTGGGT GGCTGA GCCAAG CACTTT GGCTTC TCCTCT GATCTC

5341 TTGCTC TCCTGG CTCTGG GCAGCC GCAGCG GTGCTC CGCAGG TGAGCA GCGCTG CAGCGC

5401 GGCGAT CCGGAG AGCTCA CCTCGT ACAGAA CAGTTG CAGCCA GTGGTT GGTTGC CTTAGG

5461 ATGAAG AACCCA ATGCTG GAAGCT GCGTCC CTCCTC CTGGAG AAACTG CTCCTT ATTTCC

5521 AACTTC AAGCTC TTTAGC GTGAGC GTC

The sequence of 5' miR-30E/mCxcl14[miR30-shRNA]/3' miR-30E:

1 TGTTTG AATGAG GCTTCA GTACTT TACAGA ATCGTT GCCTGC ACATCT TGGAAA CACTTG

61 CTGGGA TTACTT CGACTT CTTAAC CCAACA GAAGGC TCGAGA AGGTAT ATTGCT GTTGAC

121 AGTGAG CGACGC AGGGTC TACGAA GAATAG TAGTGA AGCCAC AGATGT ACTATT CTTCGT

181 AGACCC TGCGCT GCCTAC TGCCTC GGACTT CAAGGG GCTAGA ATTCGA GCAATT ATCTTG

241 TTTACT AAAACT GAATAC CTTGCT ATCTCT TTGATA CATTTT TACAAA GCTGAA TTAAAA

301 TGGTAT AAATTA AATCAC TTT
