# Supplementary figures and images for: ATF3‐mediated transactivation of CXCL14 in HSCs during liver fibrosis
Source: Clin Transl Med. 2024 Oct 2;14(10):e70040. doi: 10.1002/ctm2.70040 (PMC11446984; doi:10.1002/ctm2.70040)

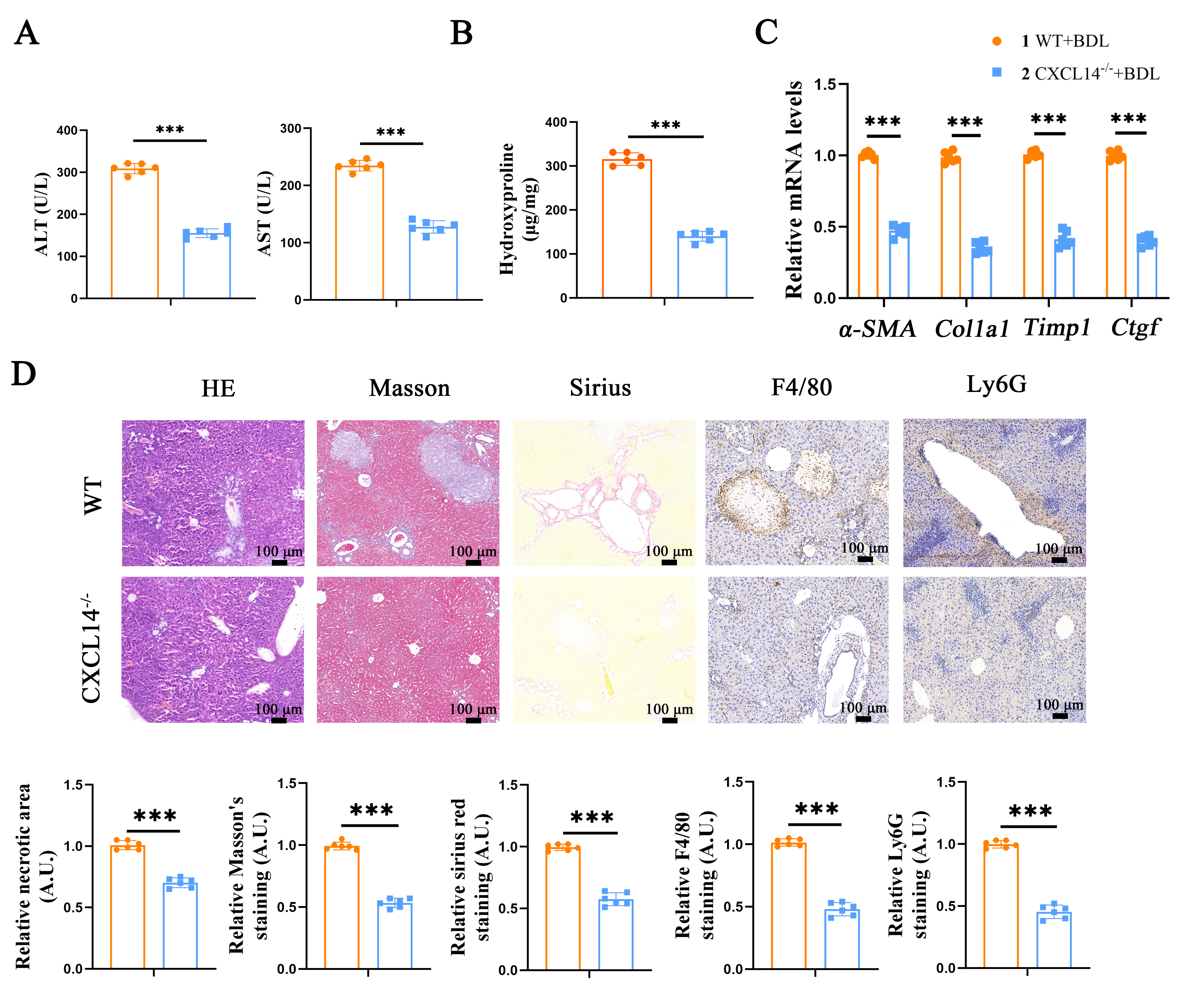

Supplement: Supplementary file 2 — Supporting information [file CTM2-14-e70040-s006.tif]

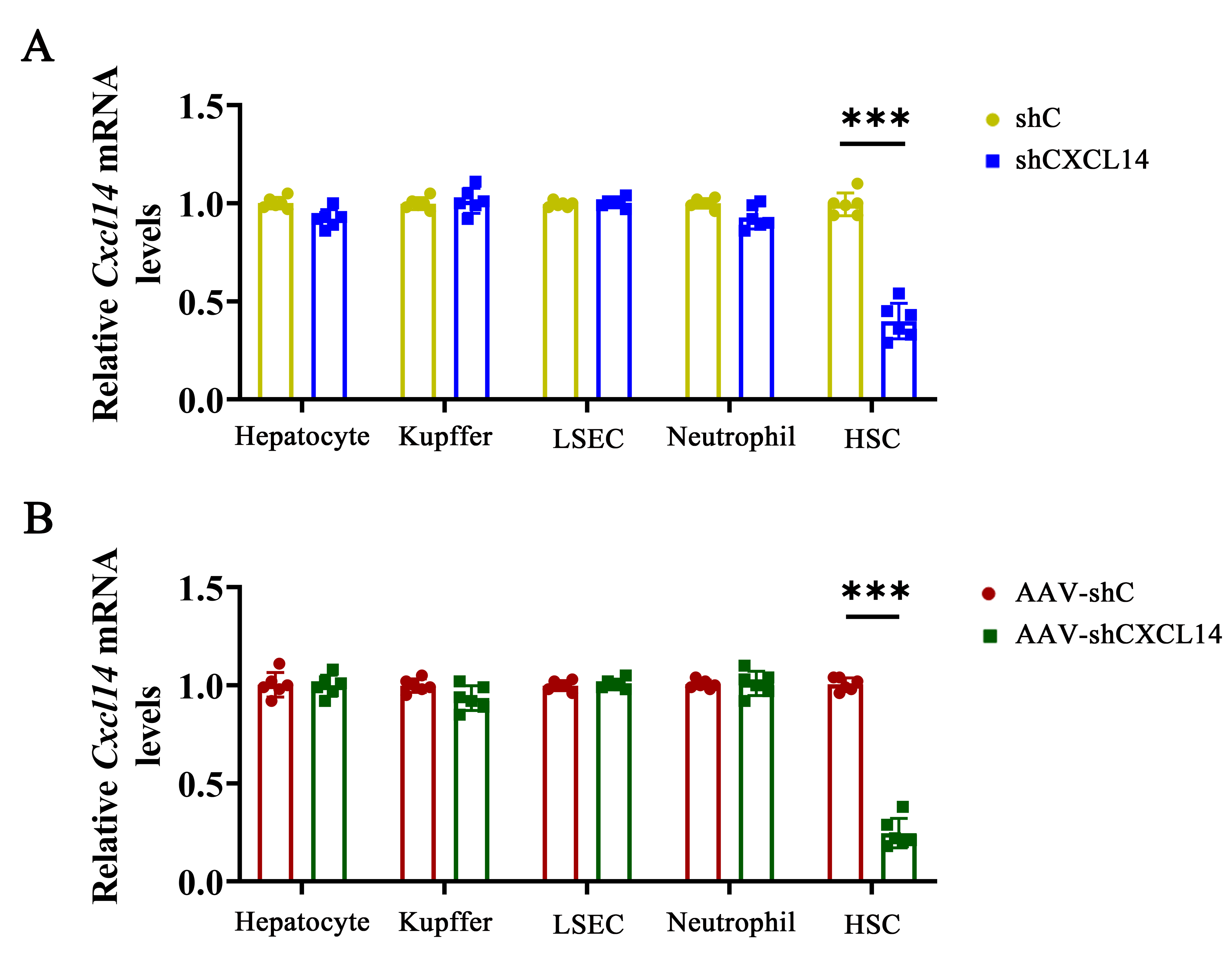

Supplement: Supplementary file 3 — Supporting information [file CTM2-14-e70040-s008.tif]

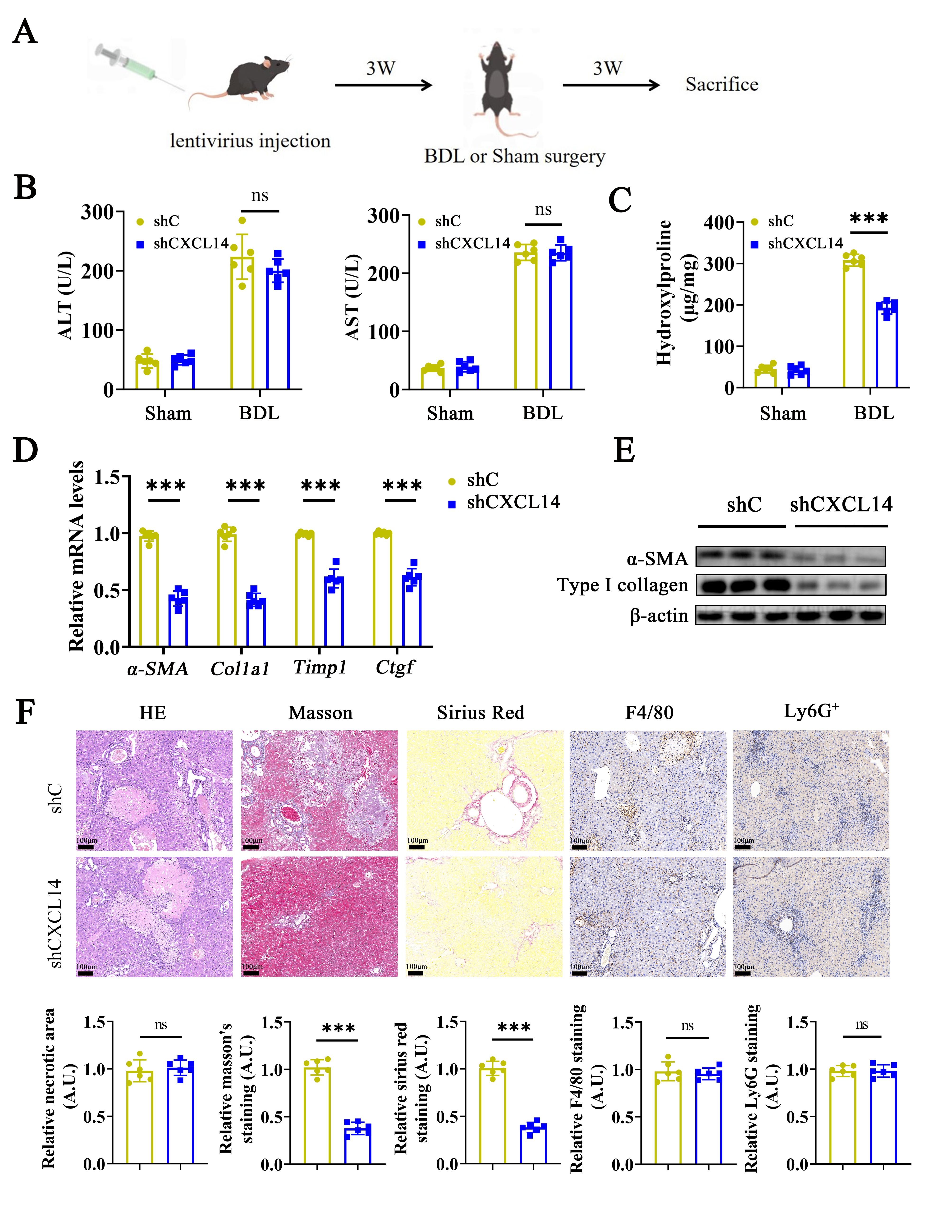

Supplement: Supplementary file 4 — Supporting information [file CTM2-14-e70040-s001.tif]

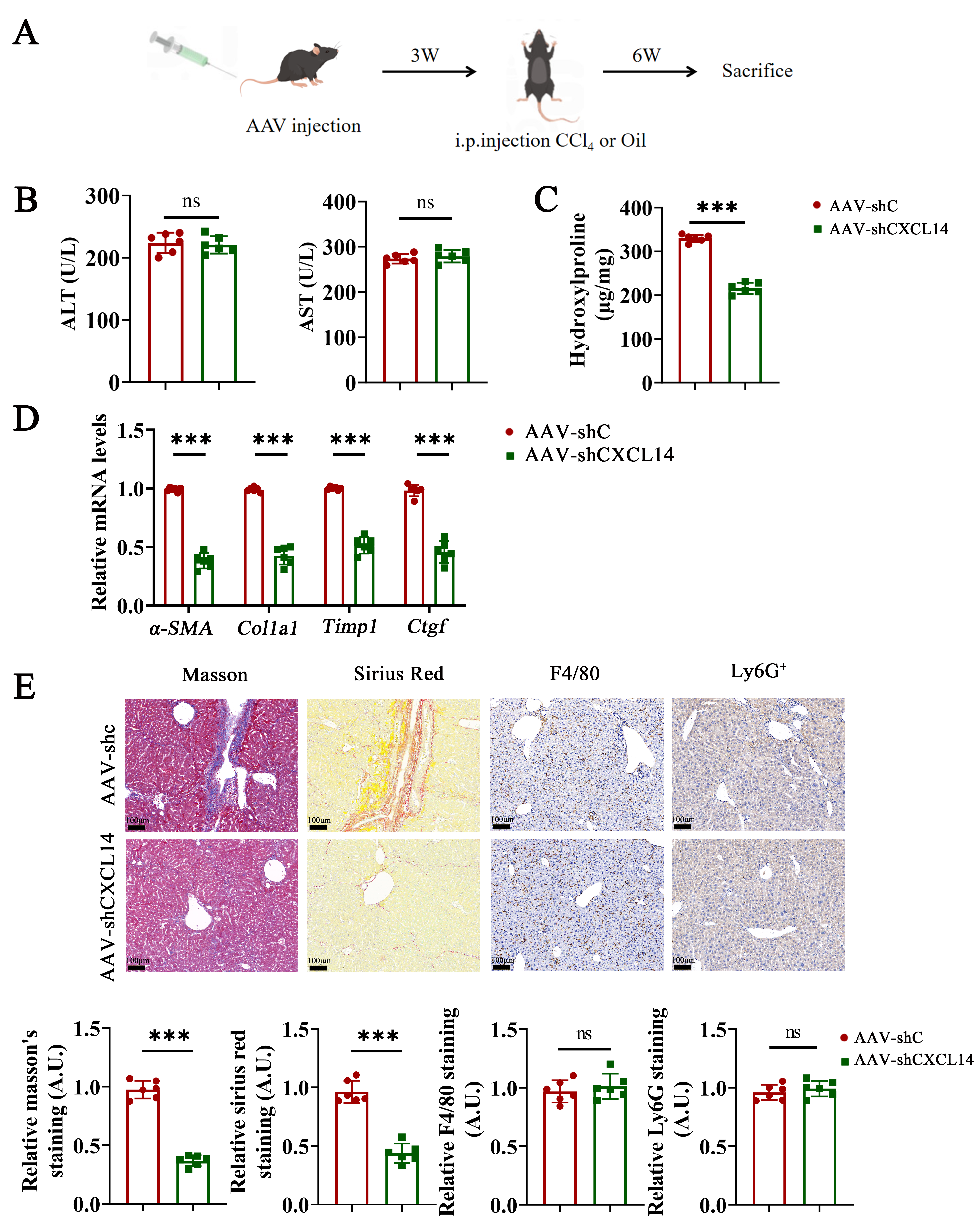

Supplement: Supplementary file 5 — Supporting information [file CTM2-14-e70040-s011.tif]

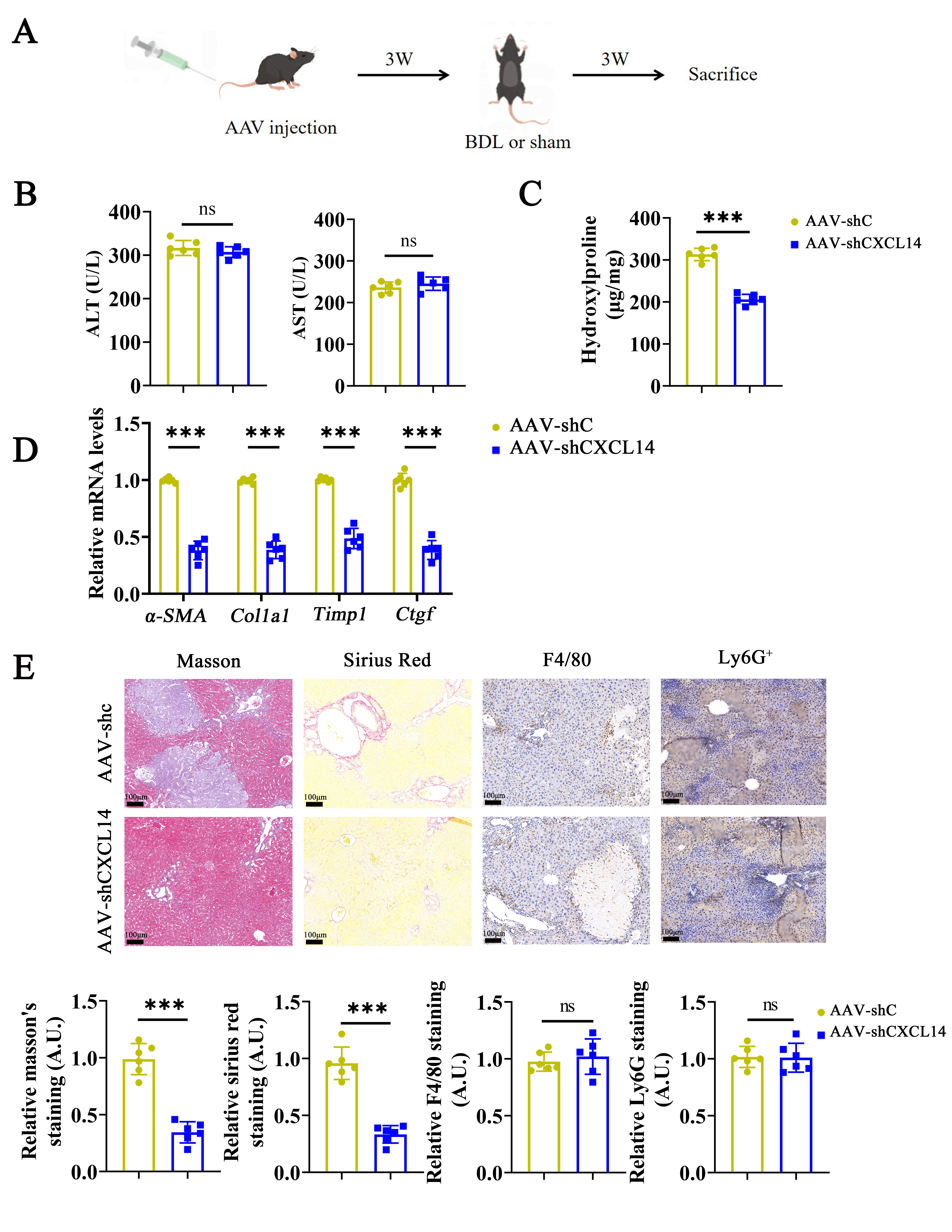

Supplement: Supplementary file 6 — Supporting information [file CTM2-14-e70040-s007.tif]

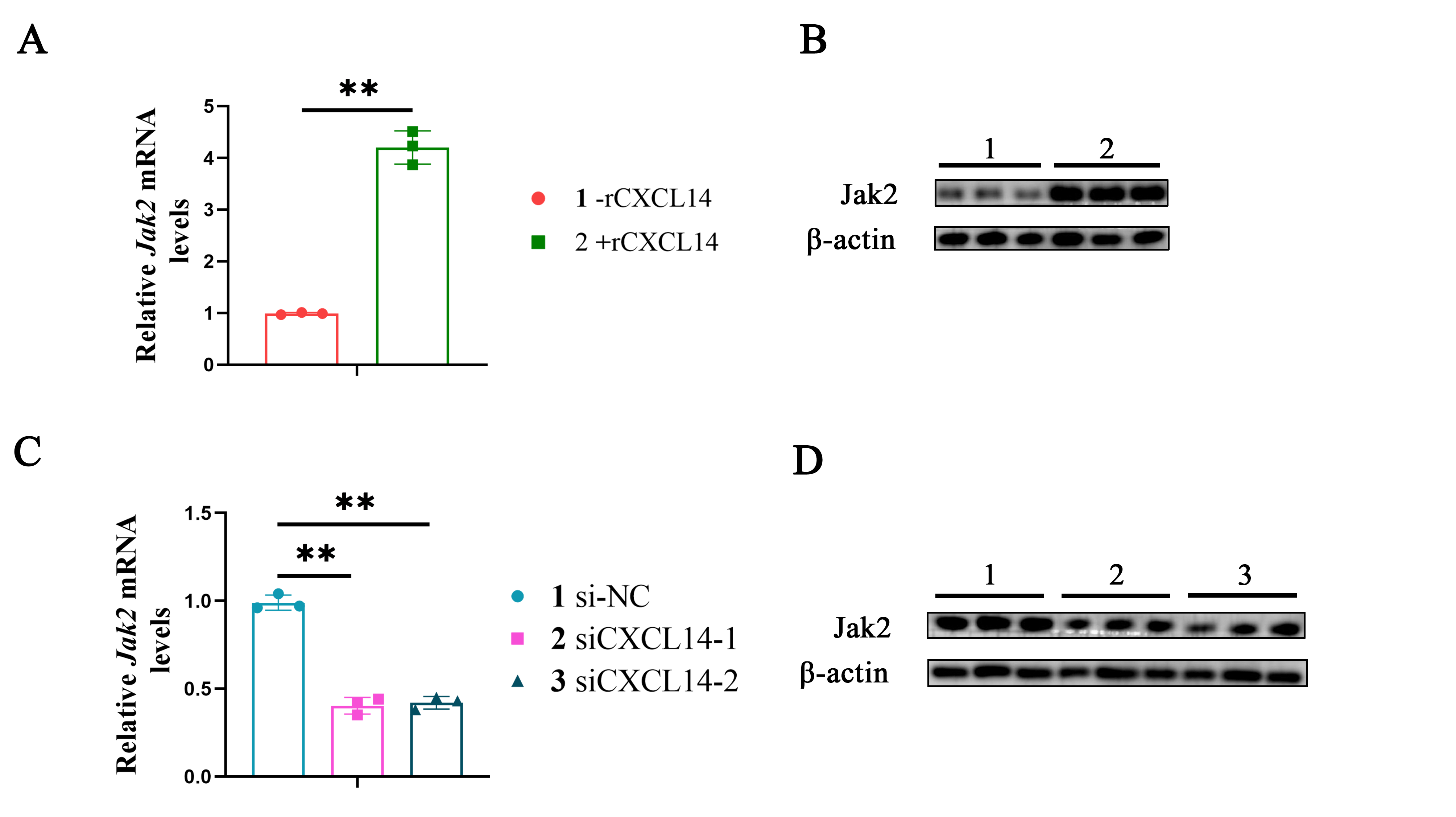

Supplement: Supplementary file 7 — Supporting information [file CTM2-14-e70040-s004.tif]

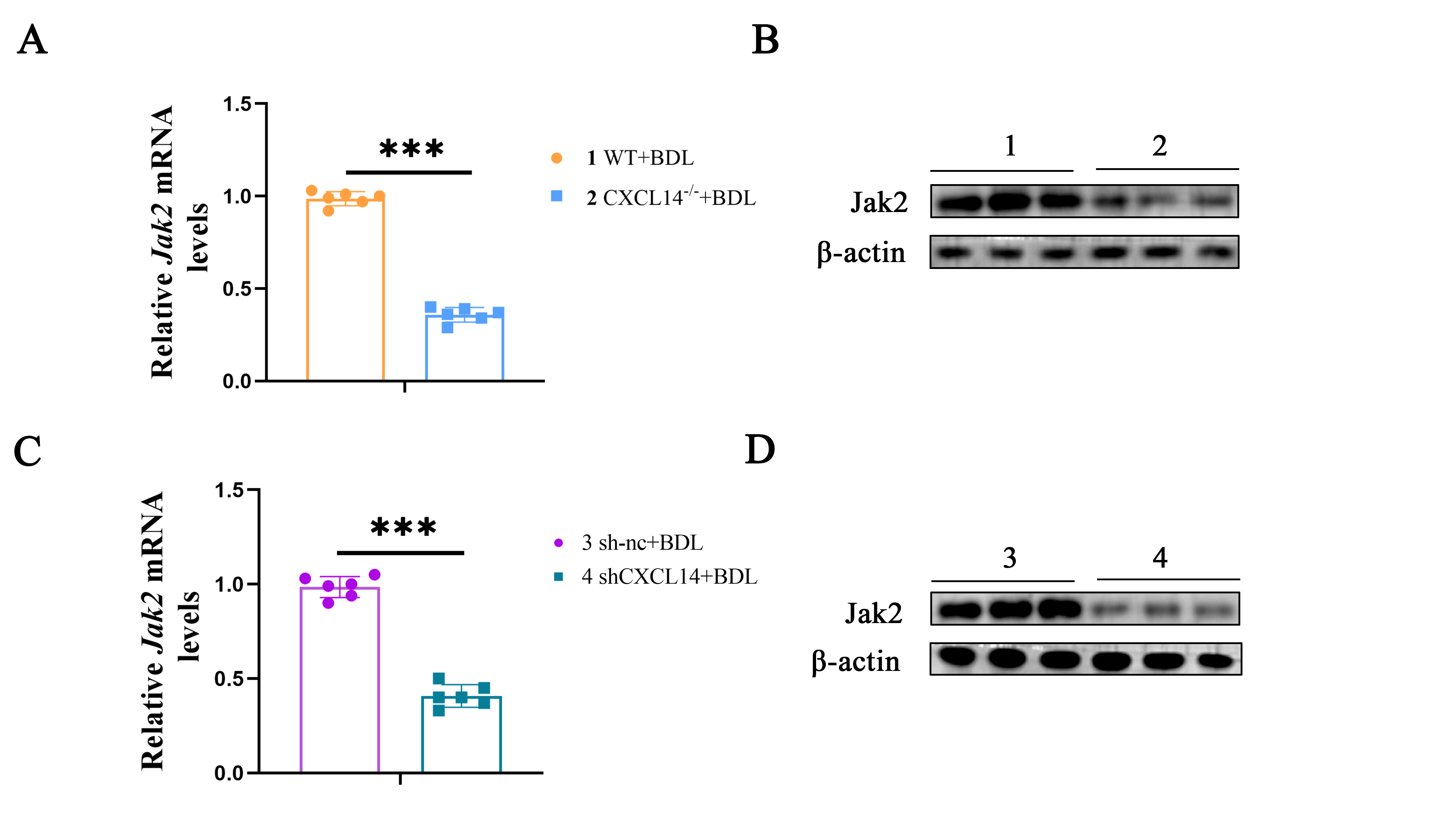

Supplement: Supplementary file 8 — Supporting information [file CTM2-14-e70040-s002.tif]

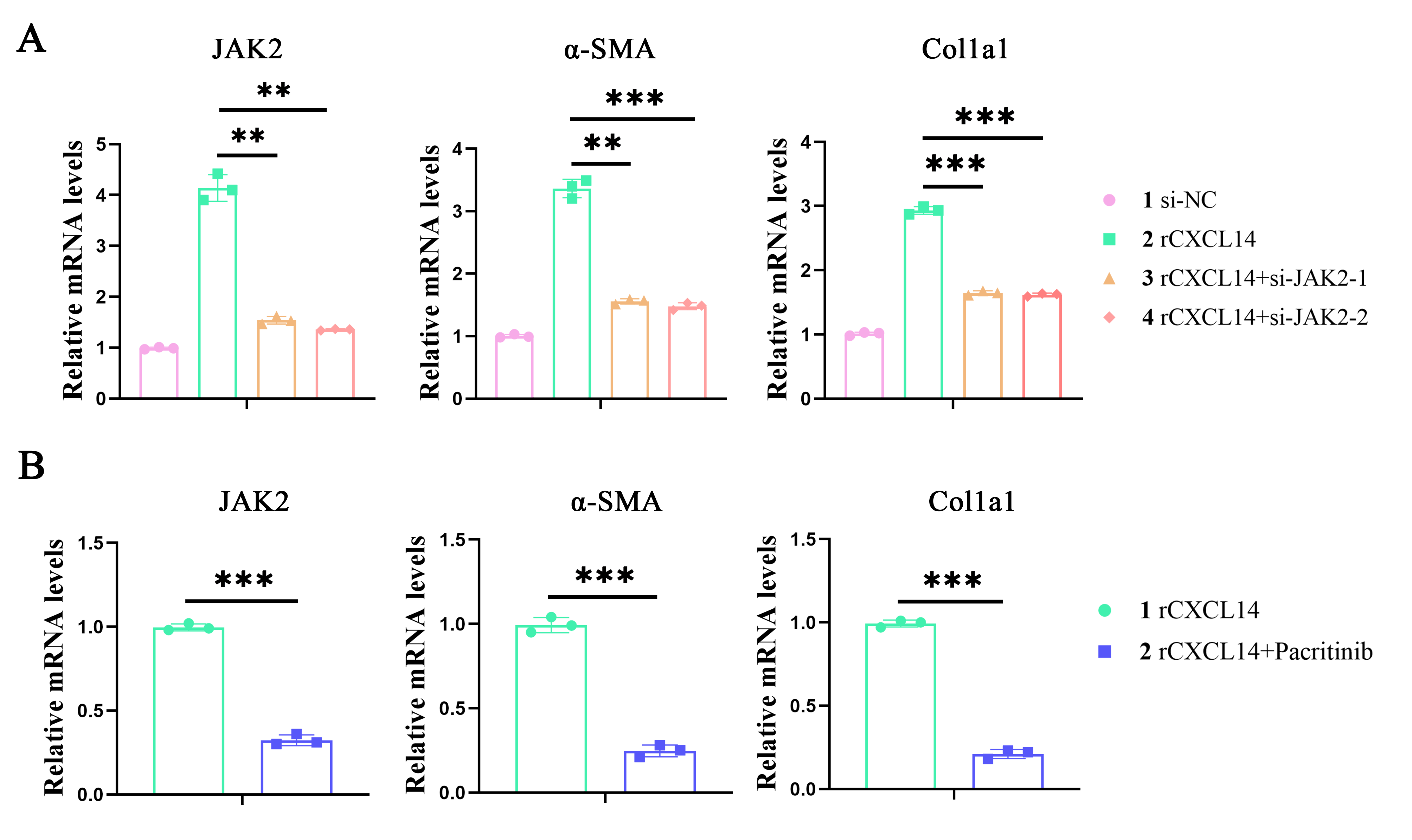

Supplement: Supplementary file 9 — Supporting information [file CTM2-14-e70040-s003.tif]

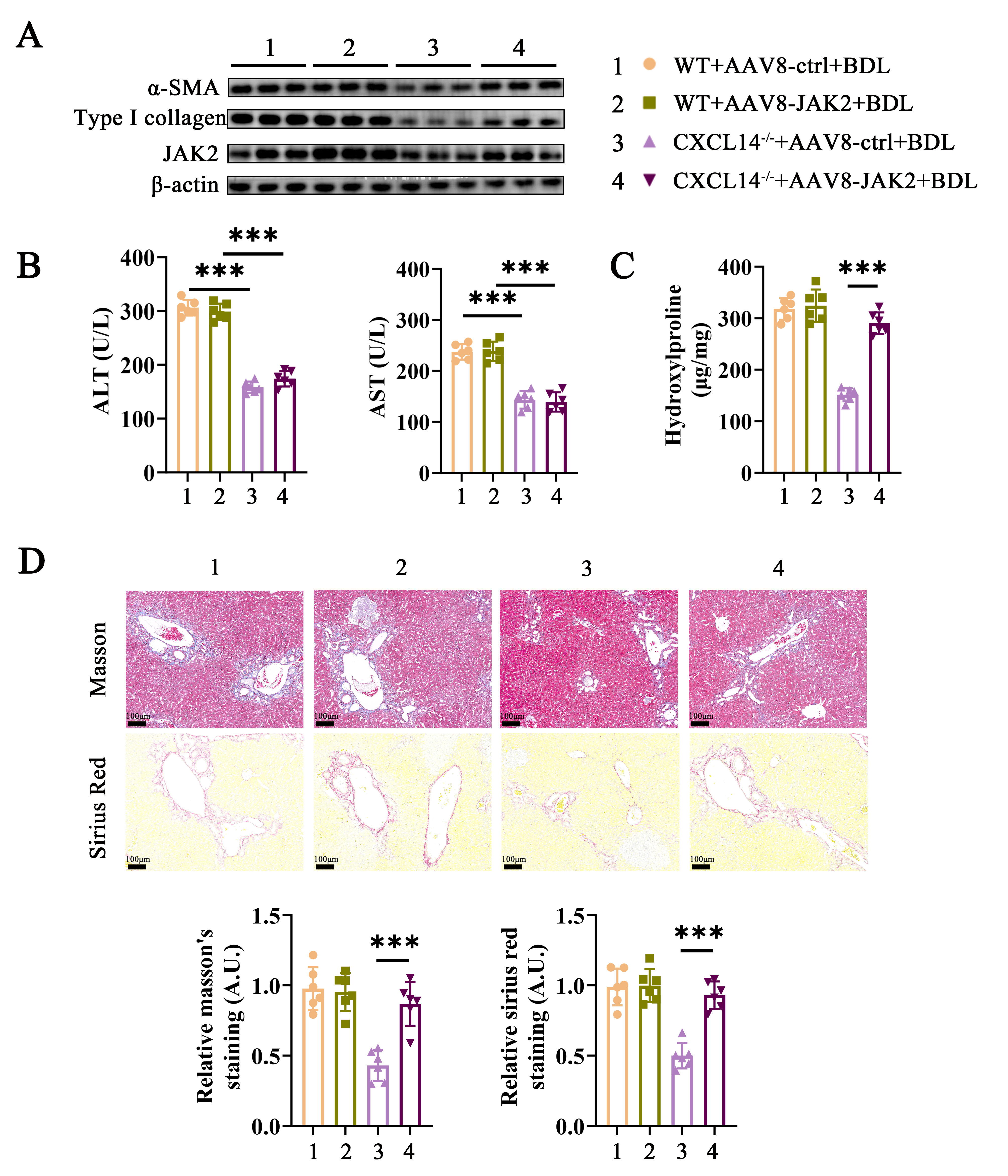

Supplement: Supplementary file 10 — Supporting information [file CTM2-14-e70040-s010.tif]

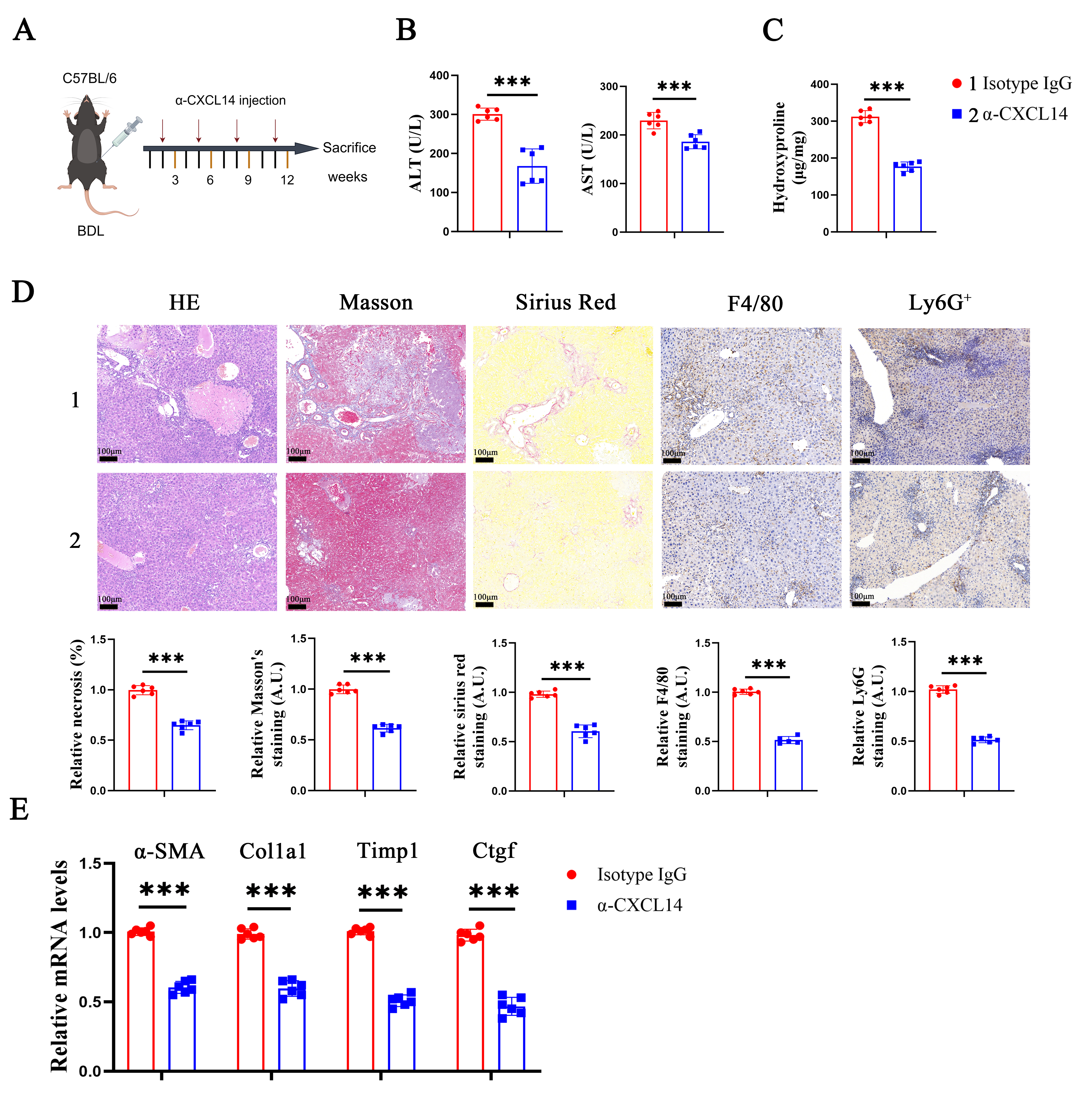

Supplement: Supplementary file 11 — Supporting information [file CTM2-14-e70040-s009.tif]
